# Supplementary material for: Gene therapy strategies for idiopathic pulmonary fibrosis: recent advances, current challenges, and future directions
Source: Mol Ther Methods Clin Dev. 2021 Jan 20;20:483–96. doi: 10.1016/j.omtm.2021.01.003 (PMC7868939; doi:10.1016/j.omtm.2021.01.003)
Supplement: Document S1. Figure S1 and Table S1 [file mmc1.pdf]

OMTM, Volume *20*

Supplemental Information

Gene therapy strategies for idiopathic pulmonary fibrosis: recent advances, current challenges, and future directions

Mitchel J.R. Ruigrok, Henderik W. Frijlink, Barbro N. Melgert, Peter Olinga, and Wouter L.J. Hinrichs

SUPPLEMENTARY INFORMATION

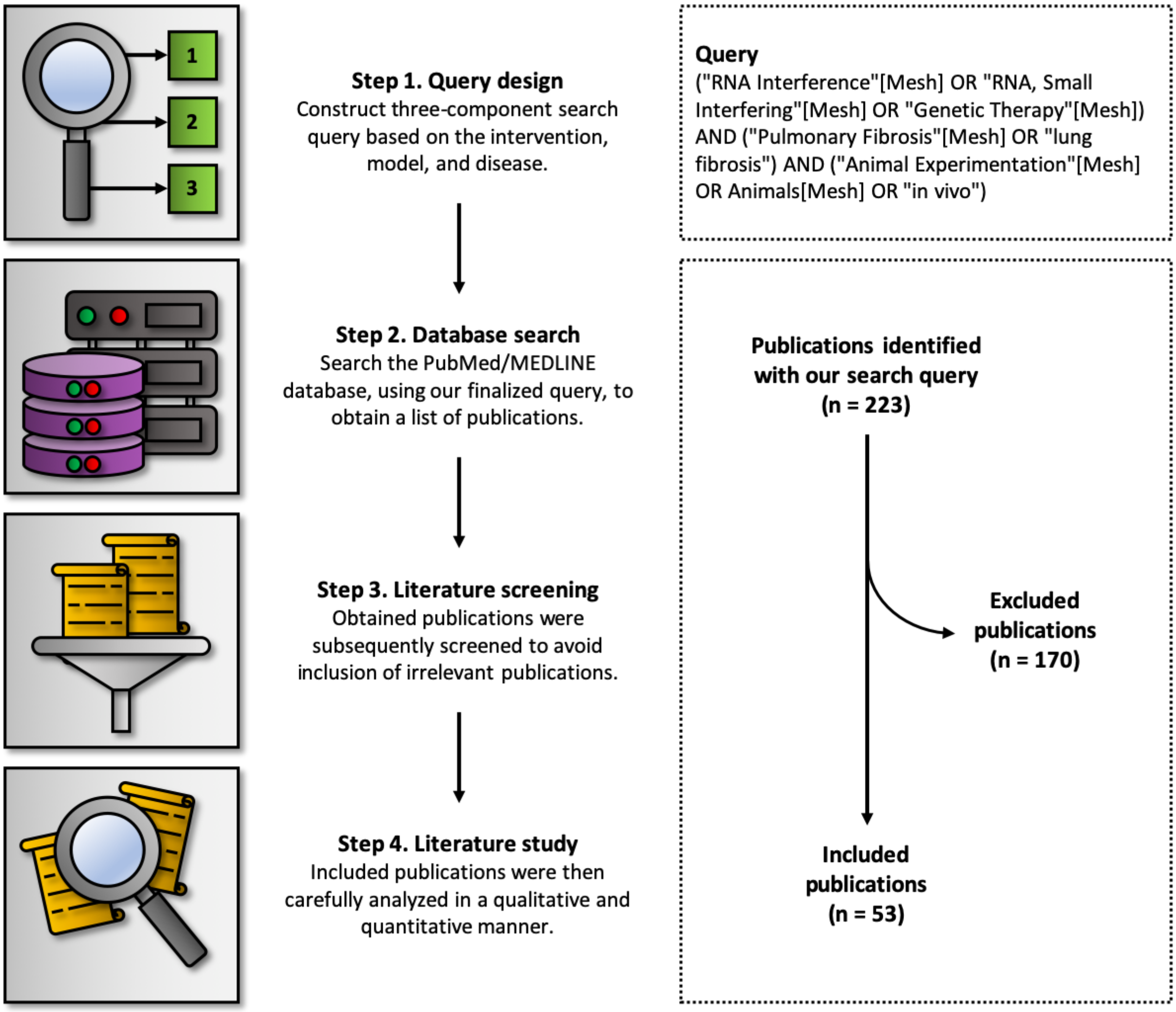

Supplemental Figure S1. Search strategy.

To find relevant publications, we conducted a literature study using the search strategy described in this figure. We first constructed a three-component search query based on the intervention (gene therapy), model (animal studies), and disease (pulmonary fibrosis). This search query was subsequently used to identify publications in the PubMed/MEDLINE database. The identified publications (n = 223) were screened to exclude publications that were not relevant (n = 170). Remaining publications (n = 53) were included in this review.

Supplemental Table S1. Nanoparticles.

This table summarizes information regarding the type and composition of nanoparticles used in cited studies. Unless not determined (n.d.), physiochemical properties are also described.

Concept	Component(s)	Cargo	Physicochemical properties	Ref.
Lipid-based	Lipofectin transfection reagent	pDNA	n.d.	[35]
Polymer-based	Macroaggregated albumin/polyethylenimine	pDNA	n.d.	[40]
Peptide-based	Hemagglutinating virus of Japan envelope proteins	siRNA	n.d.	[52]
Lipid-based	DharmaFect transfection reagent	siRNA	n.d.	[56]
Lipid-based	LipoTrust SR transfection reagent	siRNA	n.d.	[62]
Polymer-based	Poly(dimethylamino)ethylmethacrylate	siRNA	n.d.	[63]
Lipid-based	Conjugated siRNAs with lipid and polymer tails	siRNA	Z-average of ~100 nm and dispersity < 0.2	[67]
Lipid-based	Precirol and squalene	siRNA	Z-average of ~400 nm and neutral charge	[73]
Polymer-based	Polyethyleneimine	siRNA	n.d.	[78]
Peptide-based	Secondary amphipathic peptide of 20 residues	siRNA	n.d.	[79]
Polymer-based	Polyethyleneimine	pDNA	n.d.	[84]
