# Supplementary material for: Gene therapy strategies for idiopathic pulmonary fibrosis: recent advances, current challenges, and future directions
Source: Mol Ther Methods Clin Dev. 2021 Jan 20;20:483–96. doi: 10.1016/j.omtm.2021.01.003 (PMC7868939; doi:10.1016/j.omtm.2021.01.003)
Supplement: Document S2. Article plus supplemental information [file mmc2.pdf]

Molecular Therapy
Methods & Clinical Development
Review

Gene therapy strategies for idiopathic pulmonary fibrosis: recent advances, current challenges, and future directions

Mitchel J.R. Ruigrok,[1] Henderik W. Frijlink,[1] Barbro N. Melgert,[2,3] Peter Olinga,[1] and Wouter L.J. Hinrichs[1]

[1]Department of Pharmaceutical Technology and Biopharmacy, University of Groningen, Groningen Research Institute of Pharmacy, Antonius Deusinglaan 1, 9713 AV Groningen, the Netherlands; [2]Department of Molecular Pharmacology, University of Groningen, Groningen Research Institute of Pharmacy, Antonius Deusinglaan 1, 9713 AV Groningen, the Netherlands; [3]University of Groningen, Groningen Research Institute for Asthma and COPD, Hanzeplein 1, 9713 GZ Groningen, the Netherlands

Idiopathic pulmonary fibrosis (IPF) is a chronic disease in which the lungs become irreversibly scarred, leading to declining lung function. As currently available drugs do not cure IPF, there remains a great medical need for more effective treatments. Perhaps this need could be addressed by gene therapies, which offer powerful and versatile ways to attenuate a wide range of processes involved in fibrosis. Despite the potential benefits of gene therapy, no one has reviewed the current state of knowledge regarding its application for treating IPF. We therefore analyzed publications that reported the use of gene therapies to treat pulmonary fibrosis in animals, as clinical studies have not been published yet. In this review, we first provide an introduction on the pathophysiology of IPF and the most well-established gene therapy approaches. We then present a comprehensive evaluation of published animal studies, after which we provide recommendations for future research to address challenges with respect to the selection and use of animal models as well as the development of delivery vectors and dosage forms. Addressing these considerations will bring gene therapies one step closer to clinical testing and thus closer to patients.

Idiopathic pulmonary fibrosis (IPF) is a chronic and progressive disease with an unknown cause in which the lungs become irreversibly scarred. The development of IPF is largely driven by the sustained and uncontrolled activation of tissue repair mechanisms, resulting in pathological deposition of extracellular matrix (ECM) in the lungs.[1] This process often begins in both the basal and peripheral areas of the lungs. As the disease worsens, the lungs become increasingly unable to facilitate gas exchange (Figure 1). Patients will therefore experience increasing breathlessness and, eventually, respiratory failure. Currently available epidemiological studies point toward an incidence of 2–30 cases per 100,000 person-years and a prevalence of 10–60 cases per 100,000 people.[3] The prevalence, however, has been shown to increase with age. Especially older adults (>65 years) are more frequently diagnosed with IPF (494 cases per 100,000 people).[4] Most of these patients have a poor prognosis because the disease has a highly variable and unpredictable clinical course, with a median survival time of 2–3 years from the time of diagnosis.[5]

During the past few decades, numerous processes have been shown to drive wound healing and tissue repair in the lungs. Following injury, a range of cell types orchestrate various processes, such as the removal of damaged tissue by macrophages and tissue re-epithelialization by progenitor cells.[6] In IPF, however, these processes become severely dysregulated. Although the exact cause of this dysregulation remains unknown, several risk factors have been identified. Tobacco smoking, for example, is a well-recognized risk factor, especially when people smoked more than 20 pack-years (i.e., 1 pack of 20 cigarettes per day for 20 years).[7] Occupational exposure to metal, wood, and livestock-related dusts have been shown to contribute to the development of IPF as well.[8,9] Also, chronic viral infections caused by the Epstein-Barr virus, cytomegalovirus, human herpesvirus 7, and human herpesvirus 8 have been associated with IPF.[10] In some cases, patients have a genetic predisposition (i.e., familial IPF). Polymorphisms in the promotor of *MUC5B*, for instance, appear to be prognostic and are observed more often in patients suffering from IPF.[9] In the future, actual gene editing could be a promising avenue to treat patients with familial IPF. These technologies, however, are still in their infancy, so further discussion is beyond the scope of this review.

Finding novel drugs to treat IPF is challenging due to the complex pathogenesis of the disease. In fact, pinpointing a suitable molecular target is arguably the most difficult aspect. So far, only two drugs have been approved for the treatment of IPF, namely pirfenidone (Esbriet) and nintedanib (Ofev). These drugs are to be taken orally. The mechanism of action of pirfenidone remains unclear, whereas nintedanib has been shown to be a broad-spectrum tyrosine kinase inhibitor. Clinical trials have demonstrated that both drugs slow the decline in lung function by ~50% during the course of 1 year.[11,12] Although effective in slowing disease progression, these drugs do not cure IPF and may cause severe side effects (e.g., gastrointestinal bleeding and diarrhea).[1] To improve exercise tolerance in patients, clinicians also

https://doi.org/10.1016/j.omtm.2021.01.003.

Correspondence: Peter Olinga, Department of Pharmaceutical Technology and Biopharmacy, University of Groningen, Groningen Research Institute of Pharmacy, Antonius Deusinglaan 1, 9713 AV Groningen, the Netherlands.
E-mail: p.olinga@rug.nl

This is an open access article under the CC BY license (http://creativecommons.org/licenses/by/4.0/).

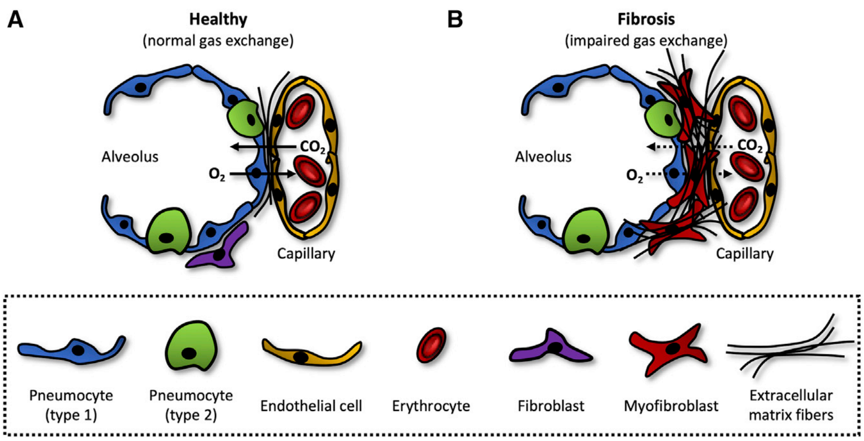

Figure 1. Gas exchange

O_2 and CO_2 diffuse across the alveolar-capillary barrier to maintain homeostasis. (A) This barrier is ~0.8 μm in healthy alveoli, enabling fast gas exchange.[2] (B) In fibrotic alveoli, the alveolar-capillary barrier is substantially thicker due to excessive deposition of ECM, thus hampering the exchange of O_2 and CO_2.

recommend oxygen supplementation and pulmonary rehabilitation, the latter of which involves exercise, education, and psychosocial support.[9] As a last resort, lung transplantation may be considered for patients, as it improves their life expectancy. Given the limited supply of donor organs and risks for graft rejection, however, many patients are not eligible for a lung transplant.[13] Clearly, treatment options are limited. More effective and safer drugs are thus greatly desired.

This need could be addressed by developing gene therapies, which hold great promise for treating a wide variety of diseases. By introducing genetic material into cells, it has become possible to modulate molecular targets previously thought to be "undruggable", a term that is often used to describe targets that cannot be regulated through conventional means (e.g., with small-molecule drugs). Despite the potential benefits of gene therapy, no one has reviewed the current state of knowledge regarding its application for treating IPF. The aim of this literature study was therefore to determine what is known about the use of gene therapy to treat pulmonary fibrosis in animals. In this review, we first provide background information on the pathophysiology of IPF and the most well-established approaches to gene therapy. We then present an evaluation of published animal studies to establish whether gene therapy could be a feasible therapeutic approach. Lastly, we discuss the challenges that need to be overcome in order to transform gene therapy concepts into drugs that benefit IPF patients.

Pathophysiology of IPF

To fathom the complex pathophysiology of IPF, it is important to be familiar with the basic principles of tissue repair. In a nutshell, the tissue repair program consists of four different, but overlapping, phases and involves a clotting/coagulation phase, an inflammation phase, a fibroblast recruitment/proliferation phase, and a remodeling phase.[14] The first phase starts directly after injury and is marked by the rapid secretion of cytokines by epithelial and endothelial cells, among others, to initiate an anti-fibrinolytic coagulation cascade that results in the formation of a temporary matrix composed of fibrin and fibronectin (FN).[15] The ensuing inflammation phase is characterized by the recruitment of various immune cells (e.g., neutrophils, macrophages, lymphocytes, and eosinophils). These cells work closely together to remove potential microbial threats and dead/damaged tissue.[16] Simultaneous secretion of cytokines sets off the third phase, in which fibroblasts are recruited. Upon activation, these fibroblasts turn into myofibroblasts, which produce a wide range of ECM proteins, especially collagen type 1.[17] In the last phase, myofibroblasts contract the wound, after which epithelial and endothelial cells are instructed to cover the freshly produced ECM.[18] Afterward, remaining myofibroblasts are instructed to undergo apoptosis or to become senescent.

In the lungs of IPF patients, the tissue repair program is severely dysregulated, favoring the excessive production of ECM proteins and the maintenance of a pro-fibrotic milieu. Up to this point, the exact cause of this dysregulation remains unknown. Emerging evidence indicates that repeated subclinical injuries to the alveolar-capillary barrier and subsequent failure to repair this barrier contribute to the development of IPF.[19] Failure to adequately repair the alveolar-capillary barrier could be caused by reduced proliferation and/or enhanced apoptosis of epithelial cells, although aberrant epithelial-mesenchymal transition (EMT) has also been shown to play a role. Somewhere in this process a point of no return is reached, resulting in the formation of a pro-fibrotic environment that hinders the resolution of fibrosis (e.g., due to epigenetic reprogramming and cellular senescence as well as changes to the matrix organization, composition, and stiffness). Thus far, a wide range of processes have been implicated in IPF, ranging from activation of the coagulation cascade to myofibroblast activation, and even auxiliary processes such as angiogenesis and oxidative stress. The extent of involvement of each process, however, has not been fully characterized yet. This makes the development of safe and effective drugs particularly challenging.

Unsurprisingly, the pathophysiology of IPF is immensely difficult to emulate in animals. In fact, there are currently no animal models capable of accurately reflecting all disease features. Animal models are therefore not specific for IPF but offer insights into some aspects of pulmonary fibrosis. Commonly used approaches to induce pulmonary fibrosis include the use of bleomycin, silica, fluorescein isothiocyanate (FITC), paraquat, or lipopolysaccharide (LPS).[20] In most cases, these substances are administered once, except for bleomycin and LPS, which may also be administered repeatedly to mimic chronic injury. These substances cause direct cell damage and/or inflammation, both of which induce a strong and robust tissue repair

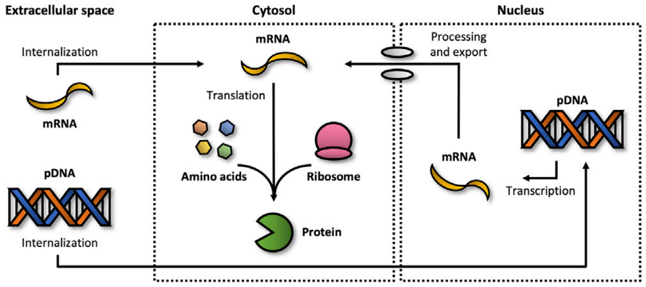

Figure 2. Enhancing expression
To restore or augment the level of a particular protein, synthetic mRNA can be delivered into the cytosol or artificially constructed pDNA can be introduced into the nucleus, producing either transient or long-term/permanent effects, respectively.

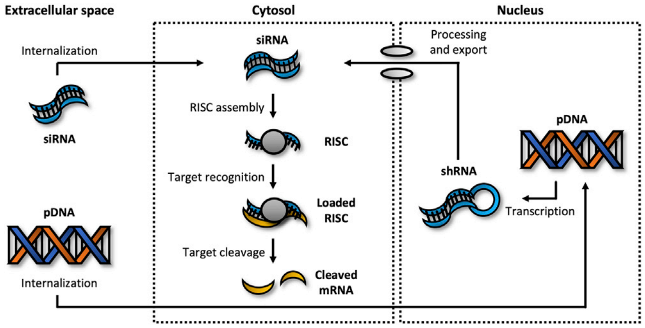

Figure 3. Silencing expression
After entering the cytosol, siRNA can induce RNAi, a process that leads to the degradation of specific target mRNA. Alternatively, pDNA-encoding shRNA can be delivered into the nucleus to achieve long-term/permanent gene silencing.

response, leading to the deposition of ECM proteins. Radiation may also be used to induce fibrosis but requires genetically susceptible (C57BL/6) mice. To avoid inducing systemic fibrosis, radiation exposure should be confined to the thoracic area using protective shields. Transgenic animals overexpressing transforming growth factor β1 (TGF-β1) are also occasionally used, allowing the animals to develop fibrosis in the absence of significant inflammation. For more information on these animal models, readers are referred to an excellent review by Moore et al.[20]

Gene therapy approaches

Enhancing expression

The first studies on gene therapy aimed to restore or augment the expression of a particular gene.[21] In the past, this aim was fulfilled by introducing artificially constructed plasmid DNA (pDNA) into the nucleus (Figure 2).[22] pDNA refers to circular, double-stranded DNA molecules that are distinct from a cell's chromosomal DNA. The use of pDNA is frequently pursued when long-term or permanent expression is desired. Vectors are required to introduce pDNA into the nucleus. Depending on the vector type, expression may persist for a long period of time or indefinitely. For example, lentiviruses (LVs) integrate pDNA into the genome of the host, resulting in permanent expression at the risk of causing insertional mutagenesis.[22] Other viral vectors, such as the adenovirus (AV) or adeno-associated virus (AAV), do not integrate pDNA into the host's genome at all (AV) or only to a limited extent (AAV), leading to long-lasting but ultimately transient expression.[23] Non-viral vectors do not result in permanent expression either; pDNA that is not integrated is eventually lost through dilution effects in dividing cells.[24]

Nowadays, gene expression may also be restored or augmented by delivering synthetic messenger RNA (mRNA) into the cytosol.[25] One of the key advantages of using mRNA is that, upon internalization by cells, it can be directly translated into protein to exert therapeutic effects intracellularly or extracellularly, without requiring further transport or processing steps. Another advantage includes the transient nature of mRNA, which reduces the risks of potential side effects. As a consequence, mRNA-based therapeutics provide greater control over the duration of effects, thus preventing continuous expression of proteins long after diseases have subsided. Despite having fewer delivery barriers than pDNA, mRNA also remains challenging to deliver due to its unfavorable physicochemical properties (e.g., large size, negative charge, and susceptibility to degradation by nucleases). As such, mRNA cannot be easily transported to and taken up by cells. Synthetic mRNAs are therefore often incorporated into non-viral vectors (e.g., nanoparticles or liposomes) for delivery and protection purposes.

Silencing expression

In 1998, Fire et al.[26] published a revolutionary paper on RNA interference (RNAi), a powerful endogenous mechanism that can be used to knock down virtually any gene. Since its discovery, RNAi has been rapidly adopted as an indispensable tool in functional genomics and drug development. To induce RNAi, small interfering RNA (siRNA) or pDNA encoding for short hairpin RNA (shRNA) has to be delivered into the cytosol or nucleus, respectively (Figure 3).[27] siRNAs are short (20–25 bp), double-stranded RNA molecules with a guide (antisense) and passenger (sense) strand. Upon entering the cytosol, siRNA is first incorporated into an RNA-induced silencing complex (RISC), after which the passenger strand is released and the guide strand retained. The activated RISC subsequently binds mRNA with a complementary sequence to the guide strand. Targeted mRNA is then cleaved and released, leading to degradation of the resulting mRNA fragments by nucleases. Afterward, the activated RISC can be reused in a new cycle to degrade another mRNA molecule.

Similar to synthetic mRNA, siRNA generally has a limited intracellular half-life of up to a few days and therefore produces only transient effects.[25,28] Non-viral vectors can be used to improve the uptake of negatively charged siRNA molecules in the lungs.[29] To achieve long-term or permanent silencing, shRNA-encoding pDNA can be delivered into the nucleus. shRNA refers to RNA molecules with a length of ~70 bp and self-complementary sites that anneal to form a tight hairpin loop.[30] However, before shRNA can induce gene silencing, additional processing steps by the ribonucleases Drosha

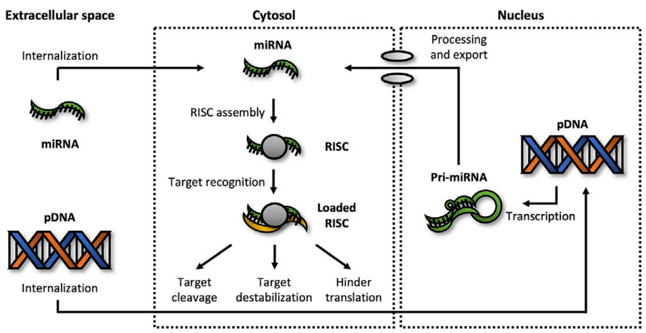

Figure 4. Repressing expression

Upon entering the cytosol, miRNA uses RNAi machinery. Depending on the sequence complementarity, target mRNA can be either cleaved or destabilized or its translation can be hindered. To achieve long-term/permanent effects, pDNA encoding pri-miRNA may be delivered into the nucleus.

and Dicer are required to turn it into siRNA. The resulting siRNA subsequently induces RNAi as described previously. Although this approach is highly effective at silencing gene expression for long periods of time, it may lead to unwanted side effects. Drosha and Dicer, for example, are also involved in the processing of endogenously expressed microRNAs (miRNAs), which regulate the expression of numerous genes. Saturation of this processing machinery could greatly affect the phenotype of cells.[31]

Repressing expression

The RNAi machinery is also used by miRNAs, which are endogenously expressed, single-stranded RNA molecules with a length of ~22 bp.[32] In the cytosol, these molecules repress the expression of genes by cleaving or destabilizing targeted mRNA or by simply hindering translation (Figure 4). Additionally, studies have revealed that expression of specific miRNAs is downregulated in various diseases, such as IPF.[33] Synthetic miRNAs therefore appear to be promising therapeutic agents. A potential benefit of miRNAs is that they often target multiple genes simultaneously, whereas siRNA targets only one specific gene. In fact, as most miRNAs display partial sequence complementarity with their corresponding mRNA, an individual miRNA could target up to 100 different mRNAs.[33] Because of this partial sequence complementarity, targeted mRNAs are rarely cleaved. Instead, while incorporated in the RNAi machinery, miRNAs either destabilize mRNA molecules by promoting deadenylation and subsequent decapping or they prevent translation by sterically hindering elongation by ribosomes.

However, despite the potential of miRNA-based therapeutics, several considerations should be taken into account. Similar to siRNA, synthetic miRNA is negatively charged, meaning its uptake can be enhanced by using non-viral vectors. Furthermore, delivering miRNA into the cytosol produces only transient effects. As an alternative, long-term or permanent expression can be achieved by introducing pDNA encoding for primary miRNA (pri-miRNA) into the nucleus. After transcription, the enzymes Drosha and Dicer, among others, are required to process pri-miRNA into miRNA. Care should be taken when selecting this approach because the pri-miRNA processing machinery could become saturated, leading to an altered expression of a number of endogenously expressed miRNAs. Although often perceived as advantageous, the pleiotropic effects of miRNA may also raise safety concerns. For that reason, target mRNA transcripts of a miRNA must be carefully mapped to avoid repressing the expression of essential genes.

Animal studies

Gene therapy clearly provides many opportunities for treating diseases. We therefore reviewed published animal studies because clinical studies have not been published yet. To find relevant publications, we searched the PubMed/MEDLINE database. See Figure S1 for more information about the literature search. We identified 53 publications, most of which described the use of a single gene therapy approach, although some reported the use of two. As illustrated, initial work in this field solely focused on enhancing gene expression for therapeutic purposes (Figure 5). In recent years, however, interests have changed in favor of gene silencing, which is currently the most frequently published approach. Repressing gene expression is reported least often, probably because miRNAs and their effects on diseases are still under thorough investigation. Nevertheless, based on the number of publications, there appears to be plenty of interest in exploring the use of gene therapy to treat IPF, regardless of the approach used. Hence this section presents key findings and highlights of the identified animal studies.

Enhancing gene expression in vivo

Seventeen publications described strategies to restore or augment expression for therapeutic purposes (Table 1). In these studies, either nanoparticles or viral vectors were used to deliver pDNA into the nuclei of cells. The use of mRNA-based therapeutics has not been described for treating pulmonary fibrosis *in vivo*. Unless stated otherwise, delivery vectors were administered intratracheally (i.t.). In one of the earliest reports, Epperly et al.[34] described that overexpression of superoxide dismutase 2 (SOD2), which catalyzes the dismutation of superoxide, protected mice from pulmonary fibrosis after irradiation. It remains unknown, however, whether this result was due to a direct effect of SOD2 on fibrosis or because SOD2 reduced oxidative stress and, subsequently, inflammation. In a follow-up study, Epperly et al.[35] further investigated the effects of SOD2 overexpression. Surprisingly, the authors observed prolonged survival but no detectable changes in tissue morphology. The discrepancy between the outcomes of these two studies is likely caused by differences in delivery vectors (adenoviruses versus liposomes), radiation doses (850–950 versus 2,000 cGy), or animal strains (nude versus C57BL/6 mice). In the same year, Nakao et al.[36] reported that bleomycin-induced fibrosis in mice could be partially suppressed by overexpressing SMAD family member 7 (SMAD7), which inhibits TGF-β signaling by blocking the formation of SMAD2/SMAD4 complexes. However, inhibiting TGF-β signaling could lead to severe side effects, such as impaired wound healing and aberrant immune activation.[51] The first attempt to treat established fibrosis was described by Sisson et al.[37] To

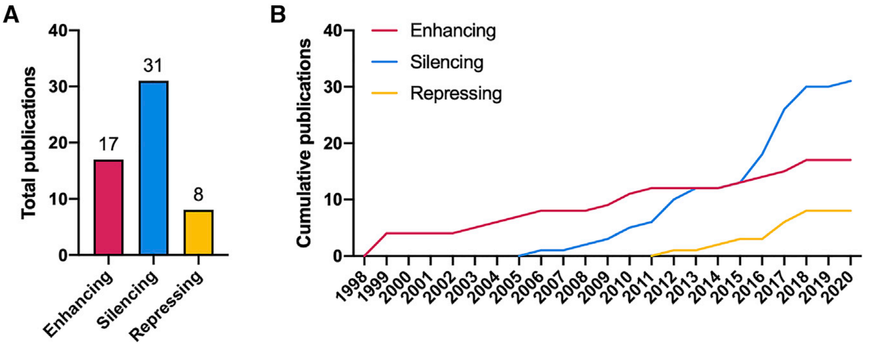

Figure 5. Publication trends

(A and B) This figure illustrates the total (A) and cumulative (B) number of publications for each gene therapy approach (i.e., enhancing, silencing, or repressing gene expression).

promote fibrinolysis in bleomycin-treated mice, they delivered adenoviruses encoding for plasminogen activator urokinase (PLAU). Although the deposition of collagen was less extensive in PLAU-overexpressing mice, fibrotic foci contained almost no expression of PLAU, indicating that the therapy prevented the progression of fibrosis instead of leading to degradation of existing collagen deposits. This observation clearly highlights the importance of verifying whether delivery vectors reach desired target sites.

A few years later, Shimizukawa et al.[38] studied whether overexpressing decorin (DCN), a proteoglycan that sequesters TGF-β, prevented bleomycin-induced fibrosis in mice. In this study, adenoviruses transduced airway and alveolar epithelial cells as well as alveolar macrophages, resulting in fewer and smaller fibrotic lesions. Empty viruses also exerted antifibrotic effects, albeit less extensive, probably due to the induction of an antiviral interferon-γ response. Around this time, an increasing interest emerged in attenuating dysregulated epithelial repair to treat fibrosis. The use of gene therapy to prevent apoptosis of epithelial cells was first studied by Inoshima et al.,[39] who described that overexpression of cyclin-dependent kinase inhibitor 1A (CDKN1) resulted in less cell death and collagen deposition in bleomycin-treated mice. Watanabe et al.[40] reported a similar approach. In this case, the authors modulated epithelial repair by restoring the expression of hepatocyte growth factor (HGF), which is a potent anti-apoptotic and mitogenic protein. They administered nanoparticles intravenously (i.v.) and observed a higher expression of HGF in the lungs and liver. HGF expression in the liver, however, was higher than that in the lungs due to interactions between nanoparticles and the hepatic mononuclear phagocyte system. Nevertheless, the therapy greatly suppressed cell death, inflammation, and fibrosis in the lungs. Although both studies showed promising results, it was not investigated whether rescued cells displayed harmful phenotypes (e.g., malignant transformation). A different approach to treat bleomycin-induced fibrosis was presented by Kijiyama et al.,[41] who inhibited activation of the coagulation cascade by augmenting tissue factor pathway inhibitor (TFPI) expression. Overexpressing TFPI suppressed various aspects of fibrosis (e.g., collagen deposition and expression of profibrotic cytokines) and almost completely eradicated procoagulant activity and thrombin generation in rats. The main disadvantage of this approach, however, is that it may lead to bleeding abnormalities, such as pulmonary hemorrhage.

Vascular remodeling affects the development of fibrosis as well. Farkas et al.,[42] for instance, demonstrated that overexpressing vascular endothelial growth factor (VEGF), which promotes angiogenesis, aggravated fibrosis in TGF-β1-transgenic rats. Therefore, Wang et al.[43] took the opposite approach and managed to alleviate bleomycin-induced fibrosis in mice by intravenously administering adenoviruses encoding for the anti-angiogenic protein vasohibin 1 (VASH1). After inhibiting angiogenesis, the authors observed less lymphocyte infiltration, cytokine secretion, and fibroblast proliferation. As the course of IPF can also be negatively impacted by pulmonary hypertension, Shenoy et al.[44] explored the effect of overexpressing angiotensin I-converting enzyme 2 (ACE2), which hydrolyzes angiotensin II (vasoconstrictor) into angiotensin (1–7) (vasodilator). In bleomycin-treated rats, this approach lowered the pulmonary arterial pressure and the deposition of collagen. Anti-angiogenic and anti-hypertensive therapies could therefore be promising for treating IPF. In the following year, Sakamoto et al.[45] reported that overexpression of fibroblast growth factor 7 (FGF7), a potent mitogenic protein, improved survival and reduced collagen deposition in mice with bleomycin-induced fibrosis. However, the authors did observe diffuse hyperplasia of FGF7-positive cells in parenchymal areas. Yang et al.[46] also studied the potential of promoting epithelial repair. To that end, the authors treated bleomycin-treated mice with inhibitor of DNA binding 2 (ID2)-encoding adenoviruses, which were administered intranasally (i.n.). They subsequently observed that ID2 overexpression stimulated proliferation of epithelial cells and lowered the extent of fibrosis. These findings are encouraging and show that promoting epithelial repair has therapeutic value, although caution is warranted when selecting delivery vectors. Lentiviruses, for example, might cause hyperplasia in the long run, as they integrate their genetic material into the host's genome, possibly resulting in incessant expression of growth factors.

More recently, Gao et al.[47] investigated whether inhibition of the interleukin 33 (IL-33)/interleukin 1 receptor-like 1 (IL-1RL1) axis could prevent bleomycin-induced fibrosis in mice. To inhibit the binding of IL-33 to its transmembrane receptor IL-1RL1, the authors intranasally administered lentiviruses encoding for soluble IL-1RL1, an isoform that actually sequesters IL-33. This approach suppressed inflammation, thereby reducing the severity of fibrosis and lowering mortality rates. A conceptually similar approach was reported by Cipolla et al.,[48] who protected mice from bleomycin-induced fibrosis by overexpressing an interleukin 17 receptor (IL-17R) fusion protein that functions as a decoy receptor for interleukin 17A (IL-17A). After

Table 1. Enhancing expression *in vivo*

Vector	Protein	Route	Treatment	Species	Model	Fibrosis	Year	Ref.
Adenovirus	SOD2	i.t.	prophylactic	mice	radiation	↓	1999	34
Nanoparticles	SOD2	i.t.	prophylactic	mice	radiation	↓	1999	35
Adenovirus	SMAD7	i.t.	prophylactic	mice	bleomycin	↓	1999	36
Adenovirus	PLAU	i.t.	therapeutic	mice	bleomycin	↓	1999	37
Adenovirus	DCN	i.t.	prophylactic	mice	bleomycin	↓	2003	38
Adenovirus	CDKN1	i.t.	prophylactic	mice	bleomycin	↓	2004	39
Nanoparticles	HGF	i.v.	prophylactic	mice	bleomycin	↓	2005	40
Adenovirus	TFPI	i.t.	prophylactic	rats	bleomycin	↓	2006	41
Adenovirus	VEGF	i.t.	prophylactic	rats	TGF-β1-Tg	↑	2009	42
Adenovirus	VASH1	i.v.	prophylactic	mice	bleomycin	↓	2010	43
Lentivirus	ACE2	i.t.	prophylactic	rats	bleomycin	↓	2010	44
Adenovirus	FGF7	i.t.	prophylactic	mice	bleomycin	↓	2011	45
Adenovirus	ID2	i.n.	prophylactic	mice	bleomycin	↓	2015	46
Lentivirus	IL-1RL1	i.n.	prophylactic	mice	bleomycin	↓	2016	47
Adenovirus	IL-17R	i.v.	prophylactic	mice	bleomycin	↓	2017	48
Adeno-associated virus	IL-10	i.t.	prophylactic	mice	bleomycin	↓	2018	49
Adeno-associated virus	TERT	i.v.	therapeutic	mice	bleomycin	↓	2018	50

Treatments were either prophylactic when administered during the onset of fibrosis or therapeutic when administered to animals with established fibrosis. Reductions in fibrosis are indicated by downward-pointing arrows (↓) and aggravations by upward-pointing arrows (↑). See Table S1 for more details on respective nanoparticles. ACE2, angiotensin I converting enzyme 2; CDKN1, cyclin-dependent kinase inhibitor 1A; DCN, decorin; FGF7, fibroblast growth factor 7; HGF, hepatocyte growth factor; ID2, inhibitor of DNA binding 2; IL-10, interleukin 10; IL-17R, interleukin 17A; IL-1RL1, interleukin 1 receptor-like 1; i.n., intranasal; i.t., intratracheal; i.v., intravenous; PLAU, plasminogen activator urokinase; SMAD7, SMAD family member 7; SOD2, superoxide dismutase 2; TERT, telomerase reverse transcriptase; TFPI, tissue factor pathway inhibitor; Tg, transgenic; TGF-β1, transforming growth factor β1; VASH1, vasohibin 1; VEGF, vascular endothelial growth factor.

administering adenoviruses intravenously, the authors observed inhibitory effects on fibrosis, apoptosis, and complement activation. It remains unknown, however, to what extent these effects were organ-specific, as the adenoviruses could have been taken up by other organs (e.g., liver and kidneys). Kurosaki et al.[49] also alleviated bleomycin-induced fibrosis by modulating inflammation. In this study, the authors augmented expression of the anti-inflammatory cytokine interleukin 10 (IL-10). While these three studies certainly present promising results, care should be taken with respect to their interpretation, as bleomycin-induced fibrosis is predominantly driven by inflammation. Because inflammation is rarely observed in IPF patients after diagnosis, such therapies may have a limited clinical relevance.[9] Unlike the previously discussed studies, Povedano et al.[50] actually managed to partially reverse established bleomycin-induced fibrosis by overexpressing telomerase reverse transcriptase (TERT) to restore the regenerative capacity of the lungs, a therapeutic approach that appears to be promising. Mice treated with intravenously administered TERT-encoding AAVs had an improved lung function and tissue morphology. Follow-up studies should characterize the tumorigenic potential of this therapeutic approach. Taken together, the publications discussed in this section demonstrate that the expression of specific genes can be enhanced to halt or ameliorate fibrosis *in vivo*.

Silencing expression in vivo

Silencing gene expression is the most frequently published approach, as evidenced by 31 publications (Table 2). About 50% of these studies reported the use of naked siRNA, 25% the use of siRNA-containing nanoparticles, and 25% the use of shRNA-expressing viral vectors. Often, these agents were administered intratracheally, unless stated otherwise. For the sake of brevity, we discuss a selection of papers that presented particularly noteworthy therapeutic approaches, study designs, or findings. In one of the first studies, Fichtner-Feigl et al.[52] revealed that silencing of interleukin 13 receptor subunit alpha 2 (IL-13RA2), which was previously thought to only serve as a decoy receptor for IL-13, protected mice from bleomycin-induced fibrosis. The authors characterized the mechanism driving this effect and discovered that IL-13 activates the TGF-β1 promotor through IL-13RA2 in a STAT6-independent and AP1-dependent manner. Apart from its therapeutic use, siRNA has also been demonstrated to be an indispensable tool in loss-of-function studies, as exemplified by Li et al.,[53] who observed more fibrotic lesions in bleomycin-treated mice upon silencing ACE2. Moreover, this study was among the first to show that locally administered naked siRNA can be used to silence gene expression in alveolar epithelial cells without using transfection reagents. The use of naked siRNA was also described by Hecker et al.,[54] who attenuated TGF-β1-induced myofibroblast differentiation as well as collagen deposition by silencing NADPH oxidase 4 (NOX4) in mice exposed to bleomycin or FITC. Immunohistochemical staining, however, revealed a lack of NOX4 silencing in fibrotic foci, indicating that naked siRNA did not diffuse into the fibrotic ECM. In the following year, Senoo et al.[55] reported that

Table 2. Silencing expression *in vivo*

Vector	Target	Route	Treatment	Species	Model	Fibrosis	Year	Ref.
Nanoparticles (siRNA)	IL-13RA2	i.t.	prophylactic	mice	bleomycin	↓	2006	52
–	ACE2	i.t.	prophylactic	mice	bleomycin	↑	2008	53
–	NOX4	i.t.	prophylactic	mice	bleomycin, FITC	↓	2009	54
–	PAI1	i.n.	prophylactic	mice	bleomycin	↓	2010	55
Nanoparticles (siRNA)	SPARC	i.t.	prophylactic	mice	bleomycin	↓	2010	56
–	CTNNB1	i.t.	prophylactic	mice	bleomycin	↓	2011	57
Adenovirus (pDNA)	SMAD3	i.t.	prophylactic	mice	paraquat	↓	2012	58
–	TGF-β1, CCL2	i.t.	prophylactic	mice	bleomycin	↓	2012	59
–	FAK	i.t.	prophylactic	mice	bleomycin	↓	2012	60
–	PAI1	i.t.	prophylactic	rats	bleomycin	↓	2012	61
Nanoparticles (siRNA)	HSP47	i.v.	therapeutic	rats	bleomycin	↓	2013	62
Nanoparticles (siRNA)	CCN2	i.t.	prophylactic	rats	bleomycin	↓	2013	63
Lentivirus (pDNA)	CTNNB1	i.t.	prophylactic	mice	silica	↓	2015	64
Lentivirus (pDNA)	JAG1	i.v.	therapeutic	mice	bleomycin	↓	2016	65
–	C3AR, C5AR	i.t.	therapeutic	mice	bleomycin	↓	2016	66
Nanoparticles (siRNA)	AREG, CCN2	i.t., i.v.	prophylactic	mice	bleomycin, TGF-β1-Tg	↓	2016	67
–	DDR2	i.n.	therapeutic	mice	bleomycin, FITC	↓	2016	68
–	CCN1	i.n.	prophylactic	mice	bleomycin	↓	2016	69
Adenovirus (pDNA)	BACH1	i.t.	prophylactic	mice	bleomycin	↓	2017	70
Adenovirus (pDNA)	FUT8	i.v.	prophylactic	mice	bleomycin	↓	2017	71
Lentivirus (pDNA)	miR-18a-5p	i.p.	prophylactic	mice	bleomycin	↑	2017	72
–	IL-17A	i.t.	therapeutic	mice	bleomycin	↓	2017	48
Nanoparticles (siRNA) + PGE2	MMP3, CCL12, HIF1A	i.t.	prophylactic	mice	bleomycin	↓	2017	73
–	CHST15	i.n.	prophylactic	mice	bleomycin	↓	2017	74
–	PDE1A	i.n.	prophylactic	rats	bleomycin	↓	2017	75
–	POSTN	i.n.	prophylactic	mice	bleomycin	↓	2017	76
Lentivirus (pDNA)	ZEB1, ZEB2	i.t.	prophylactic	mice	LPS	↓	2018	77
Nanoparticles (siRNA)	PAI1	i.t.	prophylactic	mice	bleomycin	↓	2018	78
Nanoparticles (siRNA)	SPARC, CCR2, SMAD3	i.p.	prophylactic	mice	bleomycin	↓	2018	79
–	LAMA1	i.n.	prophylactic	mice	TGF–β1-Tg	↓	2018	80
Adeno-associated virus (pDNA)	MTA1	i.p.	prophylactic	rats	bleomycin	↓	2020	81

Treatments were either prophylactic when administered during the onset of fibrosis or therapeutic when administered to animals with established fibrosis. Reductions in fibrosis are indicated by downward-pointing arrows (↓) and aggravations by upward-pointing arrows (↑). See Table S1 for more details on respective nanoparticles. AREG, amphiregulin; BACH1, BTB domain and CNC homolog 1; C3AR, complement C3a receptor 1; C5AR, complement C5a receptor 1; CCL2, C-C motif chemokine ligand 2; CCL12, C-C motif chemokine ligand 12; CCN1, cellular communication network factor 1; CCN2, cellular communication network factor 2; CCR2, C-C motif chemokine receptor 2; CHST15, carbohydrate sulfotransferase 15; CTNNB1, catenin beta 1; DDR2, discoidin domain receptor tyrosine kinase 2; FAK, focal adhesion kinase; FITC, fluorescein isothiocyanate; FUT8, fucosyltransferase 8; HIF1A, hypoxia inducible factor 1 subunit alpha; HSP47, heat shock protein 47; IL-13RA2, interleukin 13 receptor subunit alpha 2; i.p., intraperitoneal; IL-17A, interleukin 17A; JAG1, jagged canonical Notch ligand 1; LAMA1, laminin subunit alpha 1; LPS, lipopolysaccharide; MMP3, matrix metallopeptidase 3; MTA1, metastasis-associated 1; NOX4, NADPH oxidase 4; PAI1, serpin family E member 1; PDE1A, phosphodiesterase 1A; PGE2, prostaglandin E_2; POSTN, periostin; SMAD3, SMAD family member 3; SPARC, secreted protein acidic and cysteine rich; ZEB1, zinc finger E-box binding homeobox 1.

serpin family E member 1 (PAI1) silencing promoted fibrinolysis in mice with bleomycin-induced fibrosis. In this case, the authors intranasally administered naked siRNA, which was only taken up by bronchial epithelium and epithelial cells lining fibrotic foci. These studies suggest naked siRNA may be unsuitable for silencing genes that are exclusively expressed in established fibrotic lesions, possibly due to impaired diffusion.

Notwithstanding this potential delivery issue, naked siRNA can still be used to suppress the development of fibrosis in unaffected areas of the lungs. For instance, Kim et al.[57] studied whether silencing of catenin beta 1 (CTNNB1), which mediates Wnt/β-catenin signaling, conferred protection against bleomycin-induced fibrosis. Inhibiting this pathway with naked siRNA successfully reduced collagen content in the lungs of mice without affecting inflammation. Clearly, siRNA

can also be used to block other signaling pathways. Dong et al.,[58] for example, silenced SMAD3 expression to prevent TGF-β1 signaling. As a result, the authors observed fewer histopathological changes in mice with paraquat-induced fibrosis. In a follow-up to Kim et al.,[57] who showed that CTNNB1 silencing suppressed bleomycin-induced fibrosis, Wang et al.[64] conducted a study to determine whether similar effects could be observed in mice with silica-induced fibrosis. Testing therapies in different models is helpful, as pathological features differ greatly from one another (e.g., cell damage by bleomycin versus stimulation of tissue response by silica particles).[82] Ultimately, this study supports previously reported findings, as silicotic nodules were considerably smaller and less abundant in treated mice. It remains unclear, however, which cells are most affected upon silencing of CTNNB1. Some evidence indicates the involvement of pulmonary capillary endothelial cells (PCECs), as illustrated by Cao et al.,[65] who discovered that Wnt/β-catenin signaling in PCECs contributes to fibrosis by upregulating jagged canonical Notch ligand 1 (JAG1), which in turn enhances Notch signaling in nearby perivascular fibroblasts. In fact, silencing JAG1 using intravenously administered shRNA-expressing lentiviruses was shown to suppress established bleomycin-induced fibrosis in mice. Inhibiting these signaling pathways thus appears to be a powerful strategy to control the phenotype of myofibroblasts. Having said that, little is known about long-term implications; perhaps, compensation mechanisms become activated to counteract antifibrotic effects.[83]

The merit of testing gene therapies in different fibrosis models has also been demonstrated by Yoon et al.,[67] who explored whether silencing of growth factors amphiregulin (AREG) and cellular communication network factor 2 (CCN2) protected bleomycin-treated and TGF-β1-transgenic mice from fibrosis. Regardless of the fibrosis model and administration route (intranasal or intravenous), siRNA-containing nanoparticles markedly improved the morphology of lung tissue and reduced the production of COL1A1 and FN. This study also showed that, when administered intratracheally, nanoparticles were easily taken up by airway and alveolar epithelial cells, mesenchymal cells, macrophages, and T cells. Zhao et al.[68] also used two fibrosis models (i.e., established bleomycin and FITC-induced fibrosis). After silencing discoidin domain receptor tyrosine kinase 2 (DDR2) expression in mice using intranasally administered naked siRNA, the authors observed reduced myofibroblast differentiation in both models. Interestingly, because DDR2 is primarily expressed by mesenchymal cells in fibrotic lesions, this study suggests naked siRNA does diffuse into fibrotic lesions, thereby contradicting previously discussed publications. This discrepancy, however, cannot be readily explained and requires follow-up research to characterize the diffusion kinetics of naked siRNA in established fibrotic lesions. The same recommendation applies to research carried out by Kurundkar et al.,[69] who suppressed bleomycin-induced fibrosis in mice by intranasally administering naked siRNA to silence expression of the growth factor cellular communication network factor 1 (CCN1), thus inhibiting TGF-β1/SMAD2–3 signaling. CCN1 is usually expressed in areas of active fibrosis, but because the siRNA was delivered during the onset of fibrosis, it was not possible to determine whether naked siRNA could affect established fibrotic lesions. As an alternative, TGF-β1/SMAD2–3 signaling may also be inhibited by silencing the expression of fucosyltransferase 8 (FUT8), which facilitates TGF-β1 and platelet-derived growth factor subunit β (PDGFβ) activation through core fucosylation, as described by Sun et al.[71] In this study, bleomycin-treated mice were injected intravenously with shRNA-expressing adenoviruses, resulting in less collagen deposition.

More recently, therapies are being developed using multiple siRNAs, as they allow for simultaneous suppression of various pathways. On top of that, the use of multiple siRNAs in combination with an antifibrotic compound could be even more efficacious due to synergistic effects, as demonstrated by Garbuzenko et al.[73] This study elegantly showed that bleomycin-induced fibrosis in mice was suppressed more effectively by nanoparticles containing prostaglandin E_2 (PGE2) as well as siRNAs targeting matrix metallopeptidase 3 (MMP3), C-C motif chemokine ligand 12 (CCL12), and hypoxia inducible factor 1 subunit alpha (HIF1A) than by nanoparticles containing either PGE2 or siRNAs alone. An alternative approach to reduce the accumulation of myofibroblasts was tested by Ding et al.,[78] who attempted to promote fibrinolysis by silencing PAI1 while inhibiting C-X-C chemokine receptor type 4 (CXCR4)-mediated recruitment of fibrocytes. In this case, the authors encapsulated siRNA and cyclam derivatives with a high affinity for CXCR4 in nanoparticles, which exerted strong antifibrotic and anti-inflammatory effects in mice with bleomycin-induced fibrosis. Therapeutic effects, however, were mostly due to silencing of PAI1 because CXCR4-inhibiting nanoparticles alone produced only modest effects. Ding et al.[79] also developed a combinatorial therapy to treat fibrosis. Their aim was to determine whether simultaneous silencing of secreted protein acidic and cysteine rich (SPARC), C-C motif chemokine receptor 2 (CCR2), and SMAD3 could protect mice from bleomycin-induced fibrosis. siRNAs were therefore encapsulated in nanoparticles and administered intraperitoneally (i.p.). Although the authors did not characterize the biodistribution of these nanoparticles, they did observe successful silencing of respective target genes in the lungs, leading to reduced collagen deposition and inflammation. Whether simultaneous silencing of SPARC, CCR2, and SMAD3 produced synergistic effects remains unknown, as the effects of individual siRNAs were not tested. In any case, these studies show that the expression of specific genes can be silenced to attenuate a myriad of fibrosis-related processes, using either naked siRNA, siRNA-containing nanoparticles, or shRNA-expressing viral vectors.

Repressing expression in vivo

Only a few studies have examined whether miRNA supplementation is suitable for treating fibrosis (Table 3). In these studies, miRNA levels in animals were supplemented using either naked miRNA, cholesterol-conjugated miRNA, or pri-miRNA-expressing viral vectors/nanoparticles. These agents were administered intratracheally, unless specified otherwise. In one of the first reports, Xiao et al.[84] examined whether supplementation of miR-29b attenuated established bleomycin-induced fibrosis. To supplement miR-29b, the authors intravenously injected mice with pri-miRNA-encoding nanoparticles, resulting in reduced collagen deposition and macrophage infiltration. However, α-smooth muscle actin (α-SMA) expression remained unaffected.

Table 3. Repressing expression *in vivo*

Vector	miR	Route	Treatment	Species	Model	Outcome	Year	Ref.
Nanoparticles (pDNA)	29b	i.v.	therapeutic	mice	bleomycin	↓	2012	84
Cholesterol-conjugated miR	29b	i.v.	prophylactic	mice	bleomycin	↓	2014	85
Cholesterol-conjugated miR	486-5p	i.t.	prophylactic	mice	bleomycin, silica	↓	2015	86
–	503	i.t.	prophylactic	mice	silica	↓	2017	87
Lentivirus (pDNA)	18a-5p	i.p.	therapeutic	mice	bleomycin	↓	2017	72
–	30a	i.t.	prophylactic	mice	bleomycin	↓	2017	88
Lentivirus (pDNA)	200b/c	i.t.	prophylactic	mice	LPS	↓	2018	77
–	542-5p	i.v.	therapeutic	mice	silica	↓	2018	89

Treatments were either prophylactic when administered during the onset of fibrosis or therapeutic when administered to animals with established fibrosis. Reductions in fibrosis are indicated by downward-pointing arrows (↓) and aggravations by upward-pointing arrows (↑). See Table S1 for more details on respective nanoparticles.

This suggests that miR-29b affects ECM synthesis but not the accumulation of myofibroblasts. In follow-up research, Montgomery et al.[85] further studied the effects of miR-29b. In this study, bleomycin-treated mice were injected intravenously with nuclease-resistant cholesterol-conjugated miR-29b. These constructs were taken up by the lungs, where they suppressed the production of collagen and pro-inflammatory cytokines. As a next step, all miRNA-mRNA interactions should be comprehensively mapped to determine how miR-29b affects fibrosis. Cholesterol-conjugated miRNAs were also used by Ji et al.,[86] who protected mice from bleomycin and silica-induced fibrosis by supplementing miR-486-5p. Subsequent analyses revealed that miR-486-5p reduced collagen deposition at least partially by binding SMAD2 mRNA in the 3′ untranslated regions (UTRs), thereby inhibiting TGF-β1 signaling. To assess the effects of miR-503 supplementation, Yan et al.[87] administered naked miRNA to silica-treated mice and observed reduced EMT as well as fewer histopathological changes. The initiation of EMT was probably hampered due to interactions between miR-503 and the 3′ UTR of phosphatidylinositol 3-kinase (PI3K) mRNA. In addition, although the distribution of naked miR-503 in the lungs was not studied, these findings do indicate that not only naked siRNA, but also naked miRNA, is able to transfect lung cells without the use of transfection reagents.

As silencing of miR-18a-5p aggravated fibrosis *in vivo*, Zhang et al.[72] examined whether supplementing miR-18a-5p conferred protection against established bleomycin-induced fibrosis. Mice were injected intraperitoneally with pri-miRNA-encoding lentiviruses, which transduced cells in lung tissue and subsequently reduced collagen deposition by inhibiting TGF-β1/SMAD2-3 signaling. Soon after, Zhang et al.[88] reported that miR-30a supplementation suppressed myofibroblast accumulation and reduced the number of fibrotic lesions in mice that were exposed to bleomycin. However, whether miR-30a is suitable for treating fibrosis remains to be seen, as it also targets B-cell lymphoma 6 protein (BCL6), tumor suppressor p53 (P53), and runt-related transcription factor 2 (RUNX2), among others, potentially causing a wide range of side effects. The effect of miR-200b/c on fibrosis was evaluated by Cao et al.[77] Supplementing miR-200b/c in LPS-treated mice improved the visual appearance of the tissue and lowered the production of TGF-β1. EMT was also attenuated, probably because miR-200b/c regulates zinc finger E-box binding homeobox 1 (ZEB1) and ZEB2, which are known to promote EMT. Lastly, Yuan et al.[89] investigated whether intravenously injected nuclease-resistant naked miR-542-5p reversed established silica-induced fibrosis in mice. This approach effectively suppressed the production of COL1A1 and FN as well as the extent of EMT. miR-542-5p was also shown to bind to the 3′ UTR of integrin alpha 6 (ITGA6) mRNA, leading to impaired focal adhesion kinase (FAK)/PI3K/AKT signaling. Taken together, the publications discussed in this section demonstrate that miRNA-based therapeutics have great potential as they repress multiple fibrosis-related genes simultaneously. Despite these promising findings, not all miRNA-mRNA interactions have been mapped, raising safety concerns, as side effects may eventually develop. Transcriptome profiling techniques, such as next-generation sequencing, should be used more often to characterize such interactions.

Challenges and future directions

Throughout the years, considerable progress has been made regarding the application of gene therapy for treating pulmonary fibrosis *in vivo*. Indeed, this literature study confirmed that gene therapy offers exciting and promising new avenues to attenuate a wide range of processes involved in the development of fibrosis (Figure 6). Although all three gene therapy approaches (i.e., enhancing, silencing, or repressing expression) were shown to be efficacious, the use of siRNA appears to be the most promising, as it has a more favorable safety profile than miRNA and because siRNA has to cross fewer biological barriers than pDNA. At this point, it is difficult to designate a specific target that is the most suitable, as practically all of them attenuated fibrosis. This could indicate that gene therapies should target various processes simultaneously. In most cases, therapies either suppressed or halted the progression of fibrosis. In an exceptional case, however, established fibrosis was partially reversed (i.e., by augmenting the expression of TERT); follow-up studies are warranted to check whether this approach is safe. Despite these encouraging findings, we identified several challenges that should be addressed before advancing therapies to clinical trials. In this section, we discuss these challenges—which concerns the selection and use of animal models as well as the development of delivery vectors and dosage forms—and provide recommendations for future research.

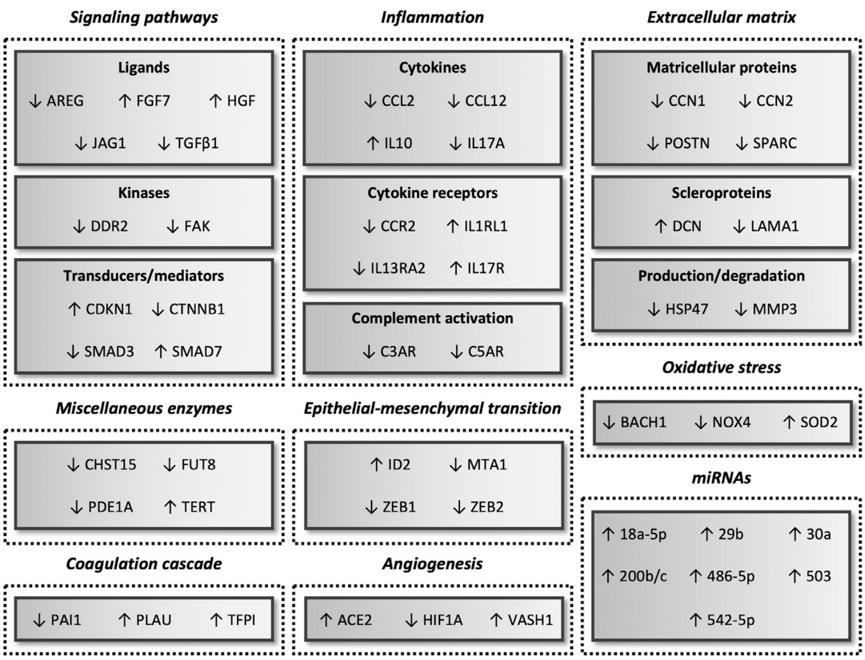

Figure 6. Successful gene therapy approaches
Enhancing gene expression and supplementing miRNA levels are denoted by upward-pointing arrows (↑) and gene silencing by downward-pointing arrows (↓).

Rethinking the selection and use of animal models

Careful selection of animal models is required to advance our understanding of proposed therapies in animals with pulmonary fibrosis. So far, the use of bleomycin is most frequently reported (Figure 7). Only a few publications describe the use of different models, and even fewer describe the use of more than one model. Although the bleomycin model has provided valuable insights into the pathogenesis of pulmonary fibrosis and potential therapeutic targets, it does not recapitulate all pathophysiological features of IPF, nor do other models address all aspects. Actually, each model displays a distinct pathological phenotype.[20,82,90] Silica-induced fibrosis, for example, is characterized by low-to-moderate infiltration of immune cells as well as the formation of nodular fibrotic lesions, whereas bleomycin- and paraquat-induced fibrosis are marked by severe inflammation and more diffuse fibrosis.[82] On top of that, paraquat is also known to cause hemorrhagic lesions. Given these differences, it is important to determine whether successful therapies also produce antifibrotic effects in other relevant non-bleomycin models. Furthermore, emerging evidence has revealed that aged mice are more susceptible to fibrosis and display impaired resolution and regeneration after injury, thus reflecting the pathogenesis of IPF more accurately.[91] Evaluating gene therapies in aged mice could therefore be a worthwhile endeavor when antifibrotic effects have already been demonstrated in young mice. However, using aged mice is not recommended in early stages of research due to considerable financial and practical hurdles associated with maintaining a cohort of aged animals.

Another problem is that most therapies were administered in a prophylactic manner before or shortly after exposing animals to fibrogenic agents, such as bleomycin (Figure 8). This is a problem, as patients are typically diagnosed with established fibrosis and because the onset of experimental fibrosis is often driven by inflammation, meaning therapeutic outcomes are more likely to be confounded by anti-inflammatory effects of therapies.[20] Bleomycin, for example, triggers severe inflammation during the first 2 weeks post-exposure. The contribution of inflammation to IPF, however, is less straightforward.[92] In fact, immunosuppressive therapies have been shown to increase mortality and hospitalization among patients, as revealed by the PANTHER-IPF trial (ClinicalTrials.gov: NCT00650091), in which a combination of prednisone, azathioprine, and *N*-acetylcysteine was compared with a placebo.[93] Furthermore, there are notable differences in molecular and histological features between early and established fibrosis; some processes are more prominent in an early stage (e.g., acute inflammation), whereas others become more prominent later on (e.g., collagen crosslinking).[94] This results in a time-dependent synthesis of ECM proteins.[95] Targeting processes that exclusively occur in early fibrosis is therefore more likely to prevent the progression of fibrosis instead of improving established fibrotic lesions. To improve their clinical translation, gene therapies should be administered to animals when inflammation has largely subsided and ECM deposition has commenced (e.g., 14–28 days after intratracheal instillation of bleomycin).[20] This enables scientists to determine whether established fibrosis is amenable to proposed gene therapies.

Developing delivery vectors and dosage forms

Once therapeutic concepts have been identified, steps can be made to develop suitable dosage forms. Pulmonary administration is clearly preferred, as it leads to site-specific delivery of genetic material in the lungs while limiting adverse effects in other organs, including the undesired accumulation of delivery vectors in the liver.[96] Local administration also vastly improves the half-life of genetic material due to the avoidance of renal clearance and nucleolytic degradation. However, before selecting inhalation devices and formulations, delivery vectors may have to be developed to ensure that genetic material is delivered (in)to targeted cells, such as macrophages, epithelial cells, or myofibroblasts. Out of all developed delivery vector technologies, ionizable lipid nanoparticles are the most clinically advanced.[97] The first-in-class siRNA-based therapeutic patisiran, sold under the brand name Onpattro, also utilizes ionizable lipid nanoparticles and was approved for medical use in the United States and European Union in 2018.[98] The advantage of ionizable lipid nanoparticles is that they can be used to efficiently transfer siRNA, miRNA, and mRNA into the cytosol of cells. Common side effects of patisiran are mild in nature and include peripheral edema and infusion-related

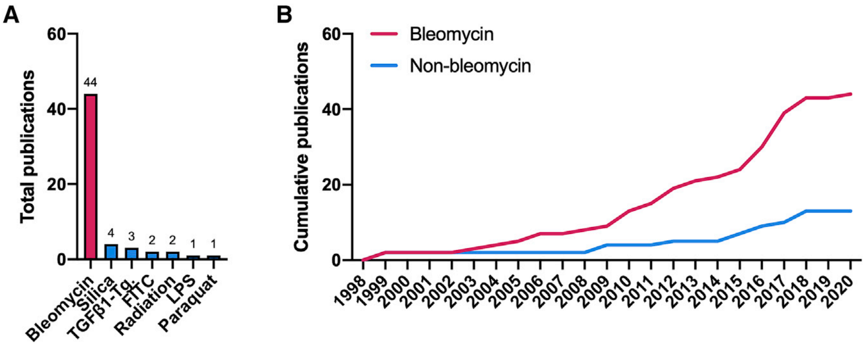

Figure 7. Animal models

(A and B) This figure depicts the total (A) and cumulative (B) number of publications for each disease model (e.g., bleomycin, radiation, silica).

reactions. While such side effects do not necessarily preclude therapeutic success, frequent administration and corresponding side effects may cause patients to either refrain from or discontinue therapy.

Depending on the type of genetic material and targeted cells, transport and uptake may be relatively easy or (extremely) difficult. Uptake of siRNA and miRNA, for instance, can occur without using delivery vectors, whereas uptake of pDNA into the nucleus cannot, thus requiring the use of nanoparticles or viral vectors. The use of delivery vectors, however, greatly affects which cells can be reached. Myofibroblasts, for example, are embedded in vast quantities of collagen-rich, tightly crosslinked ECM, which restricts the diffusion of large molecules and nano-sized structures.[15] In fact, delivery vectors with a diameter larger than 60 nm do not diffuse through dense ECM at all.[99] Contributing factors include steric interactions (collisions between genetic material and matrix proteins), hydrodynamic interactions (reduced motion of surrounding water molecules), and electrostatic interactions (attractive or repulsive forces between charged components).[100] The diffusion of naked siRNA and miRNA is probably impaired to some degree as well because these molecules were predominantly detected in bronchial epithelium but not so much within fibrotic lesions. It is therefore crucial to determine whether naked or encapsulated genetic material reaches desired target cells; clinical data are lacking and greatly desired.

Selecting inhalers also requires careful thought. Commonly used inhalers include nebulizers, soft-mist inhalers, dry powder inhalers (DPIs), and pressurized metered-dose inhalers.[96] Out of all these inhalers, DPIs are preferred for the delivery of genetic material, which is considerably more stable in a dry state than in an aqueous solution.[101] DPIs are also relatively inexpensive and effectively deposit medication in the lungs, as long as the powder particles have an aerodynamic diameter between 1 and 5 μm.[102] Nevertheless, it is currently not clear whether DPIs are suitable for patients who suffer from IPF; the delivery of powder particles to fibrotic lesions might be severely impaired due to distortions in the lung architecture. There are indications, however, that DPIs are suitable for treating IPF patients. In 2016, Galecto Biotech (Copenhagen, Denmark) successfully completed a phase 1b/2a trial (ClinicalTrials.gov: NCT02257177) to examine the safety, tolerability, and pharmacokinetics of galectin 3 inhibitor GB0139 (which was taken once daily with a DPI for 2 weeks) in healthy volunteers and IPF patients. Although results from this clinical trial have not been published yet, Galecto Biotech recently announced in a press release that the DPI formulation of GB0139 was safe and tolerated by patients. Galecto Biotech has therefore launched an international phase 2b trial (ClinicalTrials.gov: NCT03832946) to further assess the clinical efficacy and safety of GB0139 in IPF patients. Further studies are clearly required to confirm whether DPIs are indeed well tolerated by IPF patients and to determine whether dry powder formulations are deposited in the subpleural regions where fibrotic lesions are present.

Conclusions

Considerable progress has been made toward the development of gene therapies for treating IPF. This literature study confirmed that various gene therapy approaches were successfully applied *in vivo* to attenuate

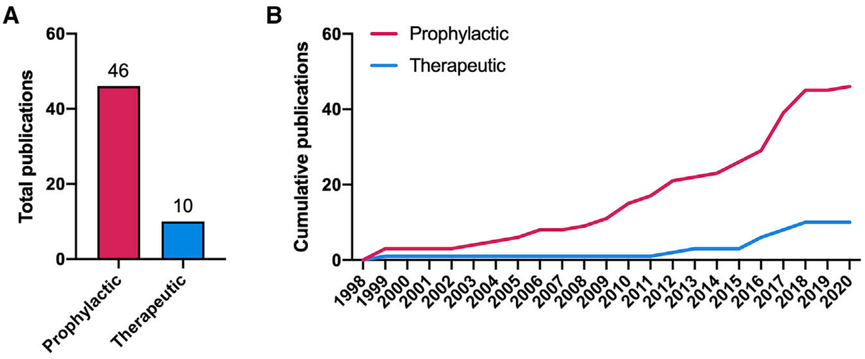

Figure 8. Treatment strategy

(A and B) This figure shows the total (A) and cumulative (B) number of publications for each treatment strategy. Treatments were prophylactic when administered during the onset of fibrosis or therapeutic when administered to animals with established fibrosis.

a wide range of fibrosis-related processes, including myofibroblast differentiation, ECM synthesis, EMT, and many more. The use of siRNA appears to be the most promising, as it has a more favorable safety profile than miRNA and because siRNA has to cross fewer biological barriers than pDNA. However, it is currently not possible to pinpoint a specific (drug) target that is most suitable, as nearly all of them attenuated fibrosis. In most cases, therapies either slowed or stopped the progression of fibrosis. In an exceptional case, however, established fibrosis was shown to be partially reversed by augmenting the expression of TERT. Despite these promising results, we identified several challenges in terms of the design of animal experiments as well as the development of delivery vectors and dosage forms. To predict therapeutic outcomes in patients with IPF more accurately, antifibrotic effects of gene therapies should be explored in different fibrosis models when inflammation has largely subsided and fibrosis has clearly commenced. In addition, it is imperative to validate whether genetic material, be it naked or formulated in delivery vectors, reaches targeted cells, especially when they are localized within fibrotic lesions. Effective therapies should preferably be administered using DPIs, as inhalation typically realizes site-specific delivery in the lungs while limiting side effects in other organs. However, as the lung architecture in IPF patients is distorted, clinical trials should be initiated to investigate whether DPIs are effective and well-tolerated. Addressing these considerations will bring potentially life-saving gene therapies one step closer to clinical trials, and thus closer to patients.

SUPPLEMENTAL INFORMATION

Supplemental Information can be found online at https://doi.org/10.1016/j.omtm.2021.01.003.

ACKNOWLEDGMENTS

The authors received no specific funding for this work.

AUTHOR CONTRIBUTIONS

M.J.R.R., H.W.F., B.N.M., P.O., and W.L.J.H. all contributed to the design, writing, and revision of the manuscript.

DECLARATION OF INTERESTS

The authors declare no competing interests.

REFERENCES

1. Lederer, D.J., and Martinez, F.J. (2018). Idiopathic pulmonary fibrosis. N. Engl. J. Med. *378*, 1811–1823.
2. Driehuys, B., Cofer, G.P., Pollaro, J., Mackel, J.B., Hedlund, L.W., and Johnson, G.A. (2006). Imaging alveolar-capillary gas transfer using hyperpolarized 129Xe MRI. Proc. Natl. Acad. Sci. USA *103*, 18278–18283.
3. Martinez, F.J., Collard, H.R., Pardo, A., Raghu, G., Richeldi, L., Selman, M., Swigris, J.J., Taniguchi, H., and Wells, A.U. (2017). Idiopathic pulmonary fibrosis. Nat. Rev. Dis. Primers *3*, 17074.
4. Raghu, G., Chen, S.Y., Yeh, W.S., Maroni, B., Li, Q., Lee, Y.C., and Collard, H.R. (2014). Idiopathic pulmonary fibrosis in US Medicare beneficiaries aged 65 years and older: incidence, prevalence, and survival, 2001–11. Lancet Respir. Med. *2*, 566–572.
5. Sgalla, G., Biffi, A., and Richeldi, L. (2016). Idiopathic pulmonary fibrosis: diagnosis, epidemiology and natural history. Respirology *21*, 427–437.
6. Hogan, B.L.M., Barkauskas, C.E., Chapman, H.A., Epstein, J.A., Jain, R., Hsia, C.C.W., Niklason, L., Calle, E., Le, A., Randell, S.H., et al. (2014). Repair and regeneration of the respiratory system: complexity, plasticity, and mechanisms of lung stem cell function. Cell Stem Cell *15*, 123–138.
7. Baumgartner, K.B., Samet, J.M., Stidley, C.A., Colby, T.V., and Waldron, J.A. (1997). Cigarette smoking: a risk factor for idiopathic pulmonary fibrosis. Am. J. Respir. Crit. Care Med. *155*, 242–248.
8. Baumgartner, K.B., Samet, J.M., Coultas, D.B., Stidley, C.A., Hunt, W.C., Colby, T.V., and Waldron, J.A.; Collaborating Centers (2000). Occupational and environmental risk factors for idiopathic pulmonary fibrosis: a multicenter case-control study. Am. J. Epidemiol. *152*, 307–315.
9. Raghu, G., Collard, H.R., Egan, J.J., Martinez, F.J., Behr, J., Brown, K.K., Colby, T.V., Cordier, J.F., Flaherty, K.R., Lasky, J.A., et al.; ATS/ERS/JRS/ALAT Committee on Idiopathic Pulmonary Fibrosis (2011). An official ATS/ERS/JRS/ALAT statement: idiopathic pulmonary fibrosis: evidence-based guidelines for diagnosis and management. Am. J. Respir. Crit. Care Med. *183*, 788–824.
10. Sheng, G., Chen, P., Wei, Y., Yue, H., Chu, J., Zhao, J., Wang, Y., Zhang, W., and Zhang, H.L. (2020). Viral infection increases the risk of idiopathic pulmonary fibrosis. Chest *157*, 1175–1187.
11. King, T.E., Jr., Bradford, W.Z., Castro-Bernardini, S., Fagan, E.A., Glaspole, I., Glassberg, M.K., Gorina, E., Hopkins, P.M., Kardatzke, D., Lancaster, L., et al.; ASCEND Study Group (2014). A phase 3 trial of pirfenidone in patients with idiopathic pulmonary fibrosis. N. Engl. J. Med. *370*, 2083–2092.
12. Richeldi, L., du Bois, R.M., Raghu, G., Azuma, A., Brown, K.K., Costabel, U., Cottin, V., Flaherty, K.R., Hansell, D.M., Inoue, Y., et al.; INPULSIS Trial Investigators (2014). Efficacy and safety of nintedanib in idiopathic pulmonary fibrosis. N. Engl. J. Med. *370*, 2071–2082.
13. Laporta Hernandez, R., Aguilar Perez, M., Lázaro Carrasco, M.T., and Ussetti Gil, P. (2018). Lung transplantation in idiopathic pulmonary fibrosis. Med. Sci. (Basel) *6*, 68.
14. Wynn, T.A. (2011). Integrating mechanisms of pulmonary fibrosis. J. Exp. Med. *208*, 1339–1350.
15. Theocharis, A.D., Skandalis, S.S., Gialeli, C., and Karamanos, N.K. (2016). Extracellular matrix structure. Adv. Drug Deliv. Rev. *97*, 4–27.
16. Lech, M., and Anders, H.-J.J. (2013). Macrophages and fibrosis: how resident and infiltrating mononuclear phagocytes orchestrate all phases of tissue injury and repair. Biochim. Biophys. Acta *1832*, 989–997.
17. Kendall, R.T., and Feghali-Bostwick, C.A. (2014). Fibroblasts in fibrosis: novel roles and mediators. Front. Pharmacol. *5*, 123.
18. Humphrey, J.D., Dufresne, E.R., and Schwartz, M.A. (2014). Mechanotransduction and extracellular matrix homeostasis. Nat. Rev. Mol. Cell Biol. *15*, 802–812.
19. Chambers, R.C., and Mercer, P.F. (2015). Mechanisms of alveolar epithelial injury, repair, and fibrosis. Ann. Am. Thorac. Soc. *12* (*Suppl 1*), S16–S20.
20. Moore, B.B., Lawson, W.E., Oury, T.D., Sisson, T.H., Raghavendran, K., and Hogaboam, C.M. (2013). Animal models of fibrotic lung disease. Am. J. Respir. Cell Mol. Biol. *49*, 167–179.
21. Friedmann, T. (1992). A brief history of gene therapy. Nat. Genet. *2*, 93–98.
22. Anguela, X.M., and High, K.A. (2019). Entering the modern era of gene therapy. Annu. Rev. Med. *70*, 273–288.
23. Kay, M.A. (2011). State-of-the-art gene-based therapies: the road ahead. Nat. Rev. Genet. *12*, 316–328.
24. Yin, H., Kauffman, K.J., and Anderson, D.G. (2017). Delivery technologies for genome editing. Nat. Rev. Drug Discov. *16*, 387–399.
25. Sahin, U., Karikó, K., and Türeci, Ö. (2014). mRNA-based therapeutics—developing a new class of drugs. Nat. Rev. Drug Discov. *13*, 759–780.
26. Fire, A., Xu, S., Montgomery, M.K., Kostas, S.A., Driver, S.E., and Mello, C.C. (1998). Potent and specific genetic interference by double-stranded RNA in *Caenorhabditis elegans*. Nature *391*, 806–811.
27. Hannon, G.J. (2002). RNA interference. Nature *418*, 244–251.
28. Bartlett, D.W., and Davis, M.E. (2006). Insights into the kinetics of siRNA-mediated gene silencing from live-cell and live-animal bioluminescent imaging. Nucleic Acids Res. *34*, 322–333.
29. Merkel, O.M., and Kissel, T. (2012). Nonviral pulmonary delivery of siRNA. Acc. Chem. Res. *45*, 961–970.

30. Rao, D.D., Vorhies, J.S., Senzer, N., and Nemunaitis, J. (2009). siRNA vs. shRNA: similarities and differences. Adv. Drug Deliv. Rev. *61*, 746–759.

31. Lam, J.K.W., Chow, M.Y.T., Zhang, Y., and Leung, S.W.S. (2015). siRNA versus miRNA as therapeutics for gene silencing. Mol. Ther. Nucleic Acids *4*, e252.

32. Rupaimoole, R., and Slack, F.J. (2017). MicroRNA therapeutics: towards a new era for the management of cancer and other diseases. Nat. Rev. Drug Discov. *16*, 203–222.

33. Ebrahimi, A., and Sadroddiny, E. (2015). MicroRNAs in lung diseases: recent findings and their pathophysiological implications. Pulm. Pharmacol. Ther. *34*, 55–63.

34. Epperly, M.W., Bray, J.A., Krager, S., Berry, L.M., Gooding, W., Engelhardt, J.F., Zwacka, R., Travis, E.L., and Greenberger, J.S. (1999). Intratracheal injection of adenovirus containing the human MnSOD transgene protects athymic nude mice from irradiation-induced organizing alveolitis. Int. J. Radiat. Oncol. Biol. Phys. *43*, 169–181.

35. Epperly, M.W., Travis, E.L., Sikora, C., and Greenberger, J.S. (1999). Manganese [correction of magnesium] superoxide dismutase (MnSOD) plasmid/liposome pulmonary radioprotective gene therapy: modulation of irradiation-induced mRNA for IL-I, TNF-alpha, and TGF-beta correlates with delay of organizing alveolitis/fibrosis. Biol. Blood Marrow Transplant. *5*, 204–214.

36. Nakao, A., Fujii, M., Matsumura, R., Kumano, K., Saito, Y., Miyazono, K., and Iwamoto, I. (1999). Transient gene transfer and expression of *Smad7* prevents bleomycin-induced lung fibrosis in mice. J. Clin. Invest. *104*, 5–11.

37. Sisson, T.H., Hattori, N., Xu, Y., and Simon, R.H. (1999). Treatment of bleomycin-induced pulmonary fibrosis by transfer of urokinase-type plasminogen activator genes. Hum. Gene Ther. *10*, 2315–2323.

38. Shimizukawa, M., Ebina, M., Narumi, K., Kikuchi, T., Munakata, H., and Nukiwa, T. (2003). Intratracheal gene transfer of decorin reduces subpleural fibroproliferation induced by bleomycin. Am. J. Physiol. Lung Cell. Mol. Physiol. *284*, L526–L532.

39. Inoshima, I., Kuwano, K., Hamada, N., Yoshimi, M., Maeyama, T., Hagimoto, N., Nakanishi, Y., and Hara, N. (2004). Induction of CDK inhibitor p21 gene as a new therapeutic strategy against pulmonary fibrosis. Am. J. Physiol. Lung Cell. Mol. Physiol. *286*, L727–L733.

40. Watanabe, M., Ebina, M., Orson, F.M., Nakamura, A., Kubota, K., Koinuma, D., Akiyama, K., Maemondo, M., Okouchi, S., Tahara, M., et al. (2005). Hepatocyte growth factor gene transfer to alveolar septa for effective suppression of lung fibrosis. Mol. Ther. *12*, 58–67.

41. Kijiyama, N., Ueno, H., Sugimoto, I., Sasaguri, Y., Yatera, K., Kido, M., Gabazza, E.C., Suzuki, K., Hashimoto, E., and Takeya, H. (2006). Intratracheal gene transfer of tissue factor pathway inhibitor attenuates pulmonary fibrosis. Biochem. Biophys. Res. Commun. *339*, 1113–1119.

42. Farkas, L., Farkas, D., Ask, K., Möller, A., Gauldie, J., Margetts, P., Inman, M., and Kolb, M. (2009). VEGF ameliorates pulmonary hypertension through inhibition of endothelial apoptosis in experimental lung fibrosis in rats. J. Clin. Invest. *119*, 1298–1311.

43. Wang, X., Zhu, H., Yang, X., Bi, Y., and Cui, S. (2010). Vasohibin attenuates bleomycin induced pulmonary fibrosis via inhibition of angiogenesis in mice. Pathology *42*, 457–462.

44. Shenoy, V., Ferreira, A.J., Qi, Y., Fraga-Silva, R.A., Díez-Freire, C., Dooies, A., Jun, J.Y., Sriramula, S., Mariappan, N., Pourang, D., et al. (2010). The angiotensin-converting enzyme 2/angiogenesis-(1–7)/Mas axis confers cardiopulmonary protection against lung fibrosis and pulmonary hypertension. Am. J. Respir. Crit. Care Med. *182*, 1065–1072.

45. Sakamoto, S., Yazawa, T., Baba, Y., Sato, H., Kanegae, Y., Hirai, T., Saito, I., Goto, T., and Kurahashi, K. (2011). Keratinocyte growth factor gene transduction ameliorates pulmonary fibrosis induced by bleomycin in mice. Am. J. Respir. Cell Mol. Biol. *45*, 489–497.

46. Yang, J., Velikoff, M., Agarwal, M., Disayabutr, S., Wolters, P.J., and Kim, K.K. (2015). Overexpression of inhibitor of DNA-binding 2 attenuates pulmonary fibrosis through regulation of c-Abl and Twist. Am. J. Pathol. *185*, 1001–1011.

47. Gao, Q., Li, Y., Pan, X., Yuan, X., Peng, X., and Li, M. (2016). Lentivirus expressing soluble ST2 alleviates bleomycin-induced pulmonary fibrosis in mice. Int. Immunopharmacol. *30*, 188–193.

48. Cipolla, E., Fisher, A.J., Gu, H., Mickler, E.A., Agarwal, M., Wilke, C.A., Kim, K.K., Moore, B.B., and Vittal, R. (2017). IL-17A deficiency mitigates bleomycin-induced complement activation during lung fibrosis. FASEB J. *31*, 5543–5556.

49. Kurosaki, F., Uchibori, R., Sehara, Y., Saga, Y., Urabe, M., Mizukami, H., Hagiwara, K., and Kume, A. (2018). AAV6-mediated IL-10 expression in the lung ameliorates bleomycin-induced pulmonary fibrosis in mice. Hum. Gene Ther. *29*, 1242–1251.

50. Povedano, J.M., Martinez, P., Serrano, R., Tejera, Á., Gómez-López, G., Bobadilla, M., Flores, J.M., Bosch, F., and Blasco, M.A. (2018). Therapeutic effects of telomerase in mice with pulmonary fibrosis induced by damage to the lungs and short telomeres. eLife *7*, 1–24.

51. Akhurst, R.J., and Hata, A. (2012). Targeting the TGFβ signalling pathway in disease. Nat. Rev. Drug Discov. *11*, 790–811.

52. Fichtner-Feigl, S., Strober, W., Kawakami, K., Puri, R.K., and Kitani, A. (2006). IL-13 signaling through the IL-13α2 receptor is involved in induction of TGF-β1 production and fibrosis. Nat. Med. *12*, 99–106.

53. Li, X., Molina-Molina, M., Abdul-Hafez, A., Uhal, V., Xaubet, A., and Uhal, B.D. (2008). Angiotensin converting enzyme-2 is protective but downregulated in human and experimental lung fibrosis. Am. J. Physiol. Lung Cell. Mol. Physiol. *295*, L178–L185.

54. Hecker, L., Vittal, R., Jones, T., Jagirdar, R., Luckhardt, T.R., Horowitz, J.C., Pennathur, S., Martinez, F.J., and Thannickal, V.J. (2009). NADPH oxidase-4 mediates myofibroblast activation and fibrogenic responses to lung injury. Nat. Med. *15*, 1077–1081.

55. Senoo, T., Hattori, N., Tanimoto, T., Furonaka, M., Ishikawa, N., Fujitaka, K., Haruta, Y., Murai, H., Yokoyama, A., and Kohno, N. (2010). Suppression of plasminogen activator inhibitor-1 by RNA interference attenuates pulmonary fibrosis. Thorax *65*, 334–340.

56. Wang, J.-C., Lai, S., Guo, X., Zhang, X., de Crombrugghe, B., Sonnylal, S., Arnett, F.C., and Zhou, X. (2010). Attenuation of fibrosis in vitro and in vivo with SPARC siRNA. Arthritis Res. Ther. *12*, R60.

57. Kim, T.H., Kim, S.-H., Seo, J.-Y., Chung, H., Kwak, H.J., Lee, S.-K., Yoon, H.J., Shin, D.H., Park, S.S., and Sohn, J.W. (2011). Blockade of the Wnt/β-catenin pathway attenuates bleomycin-induced pulmonary fibrosis. Tohoku J. Exp. Med. *223*, 45–54.

58. Dong, X.-S., Hu, X.-B., Liu, W., Sun, Y.Q., and Liu, Z. (2012). Effects of RNA interference-induced Smad3 gene silencing on pulmonary fibrosis caused by paraquat in mice. Exp. Biol. Med. (Maywood) *237*, 548–555.

59. D'Alessandro-Gabazza, C.N., Kobayashi, T., Boveda-Ruiz, D., Takagi, T., Toda, M., Gil-Bernabe, P., Miyake, Y., Yasukawa, A., Matsuda, Y., Suzuki, N., et al. (2012). Development and preclinical efficacy of novel transforming growth factor-β1 short interfering RNAs for pulmonary fibrosis. Am. J. Respir. Cell Mol. Biol. *46*, 397–406.

60. Lagares, D., Busnadiego, O., García-Fernández, R.A., Kapoor, M., Liu, S., Carter, D.E., Abraham, D., Shi-Wen, X., Carreira, P., Fontaine, B.A., et al. (2012). Inhibition of focal adhesion kinase prevents experimental lung fibrosis and myofibroblast formation. Arthritis Rheum. *64*, 1653–1664.

61. Zhang, Y.P., Li, W.B., Wang, W.L., Liu, J., Song, S.X., Bai, L.L., Hu, Y.Y., Yuan, Y.D., and Zhang, M. (2012). siRNA against plasminogen activator inhibitor-1 ameliorates bleomycin-induced lung fibrosis in rats. Acta Pharmacol. Sin. *33*, 897–908.

62. Otsuka, M., Shiratori, M., Chiba, H., Kuronuma, K., Sato, Y., Niitsu, Y., and Takahashi, H. (2017). Treatment of pulmonary fibrosis with siRNA against a collagen-specific chaperone HSP47 in vitamin A-coupled liposomes. Exp. Lung Res. *43*, 271–282.

63. Sung, D.K., Kong, W.H., Park, K., Kim, J.H., Kim, M.Y., Kim, H., and Hahn, S.K. (2013). Noncovalenly PEGylated CTGF siRNA/PDMAEMA complex for pulmonary treatment of bleomycin-induced lung fibrosis. Biomaterials *34*, 1261–1269.

64. Wang, X., Dai, W., Wang, Y., Gu, Q., Yang, D., and Zhang, M. (2015). Blocking the Wnt/β-catenin pathway by lentivirus-mediated short hairpin RNA targeting β-catenin gene suppresses silica-induced lung fibrosis in mice. Int. J. Environ. Res. Public Health *12*, 10739–10754.

65. Cao, Z., Lis, R., Ginsberg, M., Chavez, D., Shido, K., Rabbany, S.Y., Fong, G.-H., Sakmar, T.P., Rafii, S., and Ding, B.-S. (2016). Targeting of the pulmonary capillary vascular niche promotes lung alveolar repair and ameliorates fibrosis. Nat. Med. *22*, 154–162.

66. Gu, H., Fisher, A.J., Mickler, E.A., Duerson, F., 3rd, Cummings, O.W., Peters-Golden, M., Twigg, H.L., 3rd, Woodruff, T.M., Wilkes, D.S., and Vittal, R. (2016). Contribution of the anaphylatoxin receptors, C3aR and C5aR, to the pathogenesis of pulmonary fibrosis. FASEB J. *30*, 2336–2350.

67. Yoon, P.O., Park, J.W., Lee, C.-M., Kim, S.H., Kim, H.-N., Ko, Y., Bae, S.J., Yun, S., Park, J.H., Kwon, T., et al. (2016). Self-assembled micelle interfering RNA for

effective and safe targeting of dysregulated genes in pulmonary fibrosis. J. Biol. Chem. *291*, 6433–6446.

68. Zhao, H., Bian, H., Bu, X., Zhang, S., Zhang, P., Yu, J., Lai, X., Li, D., Zhu, C., Yao, L., and Su, J. (2016). Targeting of discoidin domain receptor 2 (DDR2) prevents myofibroblast activation and neovessel formation during pulmonary fibrosis. Mol. Ther. *24*, 1734–1744.
69. Kurundkar, A.R., Kurundkar, D., Rangarajan, S., Locy, M.L., Zhou, Y., Liu, R.-M., Zmijewski, J., and Thannickal, V.J. (2016). The matricellular protein CCN1 enhances TGF-β1/SMAD3-dependent profibrotic signaling in fibroblasts and contributes to fibrogenic responses to lung injury. FASEB J. *30*, 2135–2150.
70. Liu, Y., and Zheng, Y. (2017). Bach1 siRNA attenuates bleomycin-induced pulmonary fibrosis by modulating oxidative stress in mice. Int. J. Mol. Med. *39*, 91–100.
71. Sun, W., Tang, H., Gao, L., Sun, X., Liu, J., Wang, W., Wu, T., and Lin, H. (2017). Mechanisms of pulmonary fibrosis induced by core fucosylation in pericytes. Int. J. Biochem. Cell Biol. *88*, 44–54.
72. Zhang, Q., Ye, H., Xiang, F., Song, L.-J., Zhou, L.-L., Cai, P.-C., Zhang, J.-C., Yu, F., Shi, H.-Z., Su, Y., et al. (2017). miR-18a-5p inhibits sub-pleural pulmonary fibrosis by targeting TGF-β receptor II. Mol. Ther. *25*, 728–738.
73. Garbuzenko, O.B., Ivanova, V., Kholodovych, V., Reimer, D.C., Reuhl, K.R., Yurkow, E., Adler, D., and Minko, T. (2017). Combinatorial treatment of idiopathic pulmonary fibrosis using nanoparticles with prostaglandin E and siRNA(s). Nanomedicine (Lond.) *13*, 1983–1992.
74. Kai, Y., Tomoda, K., Yoneyama, H., Kitabatake, M., Nakamura, A., Ito, T., Yoshikawa, M., and Kimura, H. (2017). Silencing of carbohydrate sulfotransferase 15 hinders murine pulmonary fibrosis development. Mol. Ther. Nucleic Acids *6*, 163–172.
75. Ren, L., Yang, C., Dou, Y., Zhan, R., Sun, Y., and Yu, Y. (2017). miR-541-5p regulates lung fibrosis by targeting cyclic nucleotide phosphodiesterase 1A. Exp. Lung Res. *43*, 249–258.
76. Tomaru, A., Kobayashi, T., Hinneh, J.A., Baffour Tonto, P., D'Alessandro-Gabazza, C.N., Fujimoto, H., Fujiwara, K., Takahashi, Y., Ohnishi, M., Yasuma, T., et al. (2017). Oligonucleotide-targeting periostin ameliorates pulmonary fibrosis. Gene Ther. *24*, 706–716.
77. Cao, Y., Liu, Y., Ping, F., Yi, L., Zeng, Z., and Li, Y. (2018). miR-200b/c attenuates lipopolysaccharide-induced early pulmonary fibrosis by targeting ZEB1/2 via p38 MAPK and TGF-β/smad3 signaling pathways. Lab. Invest. *98*, 339–359.
78. Ding, L., Zhu, C., Yu, F., Wu, P., Chen, G., Ullah, A., Wang, K., Sun, M., Li, J., and Oupický, D. (2018). Pulmonary delivery of polyplexes for combined PAI-1 gene silencing and CXCR4 inhibition to treat lung fibrosis. Nanomedicine (Lond.) *14*, 1765–1776.
79. Ding, W., Pu, W., Jiang, S., Ma, Y., Liu, Q., Wu, W., Chu, H., Zou, H., Jin, L., Wang, J., and Zhou, X. (2018). Evaluation of the antifibrotic potency by knocking down SPARC, CCR2 and SMAD3. EBioMedicine *38*, 238–247.
80. Lee, C.M., Cho, S.J., Cho, W.K., Park, J.W., Lee, J.H., Choi, A.M., Rosas, I.O., Zheng, M., Peltz, G., Lee, C.G., et al. (2018). Laminin α1 is a genetic modifier of TGF-β1-stimulated pulmonary fibrosis. JCI Insight *3*, e99574.
81. Qian, W., Cai, X., Qian, Q., Zhang, W., and Tian, L. (2020). Metastasis-associated protein 1 promotes epithelial-mesenchymal transition in idiopathic pulmonary fibrosis by up-regulating Snail expression. J. Cell. Mol. Med. *24*, 5998–6007.
82. Dong, J., Yu, X., Porter, D.W., Battelli, L.A., Kashon, M.L., and Ma, Q. (2016). Common and distinct mechanisms of induced pulmonary fibrosis by particulate and soluble chemical fibrogenic agents. Arch. Toxicol. *90*, 385–402.
83. Pai, S.G., Carneiro, B.A., Mota, J.M., Costa, R., Leite, C.A., Barroso-Sousa, R., Kaplan, J.B., Chae, Y.K., and Giles, F.J. (2017). Wnt/beta-catenin pathway: modulating anticancer immune response. J. Hematol. Oncol. *10*, 101.
84. Xiao, J., Meng, X.M., Huang, X.R., Chung, A.C.K., Feng, Y.-L., Hui, D.S.C., Yu, C.-M., Sung, J.J.Y., and Lan, H.Y. (2012). miR-29 inhibits bleomycin-induced pulmonary fibrosis in mice. Mol. Ther. *20*, 1251–1260.
85. Montgomery, R.L., Yu, G., Latimer, P.A., Stack, C., Robinson, K., Dalby, C.M., Kaminski, N., and van Rooij, E. (2014). MicroRNA mimicry blocks pulmonary fibrosis. EMBO Mol. Med. *6*, 1347–1356.
86. Ji, X., Wu, B., Fan, J., Han, R., Luo, C., Wang, T., Yang, J., Han, L., Zhu, B., Wei, D., et al. (2015). The anti-fibrotic effects and mechanisms of microRNA-486-5p in pulmonary fibrosis. Sci. Rep. *5*, 14131.
87. Yan, W., Wu, Q., Yao, W., Li, Y., Liu, Y., Yuan, J., Han, R., Yang, J., Ji, X., and Ni, C. (2017). miR-503 modulates epithelial-mesenchymal transition in silica-induced pulmonary fibrosis by targeting PI3K p85 and is sponged by lncRNA MALAT1. Sci. Rep. *7*, 11313.
88. Zhang, S., Liu, H., Liu, Y., Zhang, J., Li, H., Liu, W., Cao, G., Xv, P., Zhang, J., Lv, C., and Song, X. (2017). miR-30a as potential therapeutics by targeting TET1 through regulation of Drp-1 promoter hydroxymethylation in idiopathic pulmonary fibrosis. Int. J. Mol. Sci. *18*, 633.
89. Yuan, J., Li, P., Pan, H., Li, Y., Xu, Q., Xu, T., Ji, X., Liu, Y., Yao, W., Han, L., et al. (2018). miR-542-5p attenuates fibroblast activation by targeting integrin α6 in silica-induced pulmonary fibrosis. Int. J. Mol. Sci. *19*, 3717.
90. Tashiro, J., Rubio, G.A., Limper, A.H., Williams, K., Elliot, S.J., Ninou, I., Aidinis, V., Tzouvelekis, A., and Glassberg, M.K. (2017). Exploring animal models that resemble idiopathic pulmonary fibrosis. Front. Med. (Lausanne) *4*, 118.
91. Jenkins, R.G., Moore, B.B., Chambers, R.C., Eickelberg, O., Königshoff, M., Kolb, M., Laurent, G.J., Nanthakumar, C.B., Olman, M.A., Pardo, A., et al.; ATS Assembly on Respiratory Cell and Molecular Biology (2017). An official American Thoracic Society workshop report: use of animal models for the preclinical assessment of potential therapies for pulmonary fibrosis. Am. J. Respir. Cell Mol. Biol. *56*, 667–679.
92. Heukels, P., Moor, C.C., von der Thüsen, J.H., Wijsenbeek, M.S., and Kool, M. (2019). Inflammation and immunity in IPF pathogenesis and treatment. Respir. Med. *147*, 79–91.
93. Raghu, G., Anstrom, K.J., King, T.E., Jr., Lasky, J.A., and Martinez, F.J.; Idiopathic Pulmonary Fibrosis Clinical Research Network (2012). Prednisone, azathioprine, and *N*-acetylcysteine for pulmonary fibrosis. N. Engl. J. Med. *366*, 1968–1977.
94. King, T.E., Jr., Pardo, A., and Selman, M. (2011). Idiopathic pulmonary fibrosis. Lancet *378*, 1949–1961.
95. Decaris, M.L., Gatmaitan, M., FlorCruz, S., Luo, F., Li, K., Holmes, W.E., Hellerstein, M.K., Turner, S.M., and Emson, C.L. (2014). Proteomic analysis of altered extracellular matrix turnover in bleomycin-induced pulmonary fibrosis. Mol. Cell. Proteomics *13*, 1741–1752.
96. Ruigrok, M.J.R., Frijlink, H.W., and Hinrichs, W.L.J. (2016). Pulmonary administration of small interfering RNA: the route to go? J. Control. Release *235*, 14–23.
97. Samaridou, E., Heyes, J., and Lutwyche, P. (2020). Lipid nanoparticles for nucleic acid delivery: current perspectives. Adv. Drug Deliv. Rev. *154–155*, 37–63.
98. Adams, D., Gonzalez-Duarte, A., O'Riordan, W.D., Yang, C.-C., Ueda, M., Kristen, A.V., Tournev, I., Schmidt, H.H., Coelho, T., Berk, J.L., et al. (2018). Patisiran, an RNAi therapeutic, for hereditary transthyretin amyloidosis. N. Engl. J. Med. *379*, 11–21.
99. Barua, S., and Mitragotri, S. (2014). Challenges associated with penetration of nanoparticles across cell and tissue barriers: a review of current status and future prospects. Nano Today *9*, 223–243.
100. Stylianopoulos, T., Poh, M.Z., Insin, N., Bawendi, M.G., Fukumura, D., Munn, L.L., and Jain, R.K. (2010). Diffusion of particles in the extracellular matrix: the effect of repulsive electrostatic interactions. Biophys. J. *99*, 1342–1349.
101. Chow, M.Y., and Lam, J.K. (2015). Dry powder formulation of plasmid DNA and siRNA for inhalation. Curr. Pharm. Des. *21*, 3854–3866.
102. de Boer, A.H., Hagedoorn, P., Hoppentocht, M., Buttini, F., Grasmeijer, F., and Frijlink, H.W. (2017). Dry powder inhalation: past, present and future. Expert Opin. Drug Deliv. *14*, 499–512.

OMTM, Volume *20*

Supplemental Information

Gene therapy strategies for idiopathic pulmonary fibrosis: recent advances, current challenges, and future directions

Mitchel J.R. Ruigrok, Henderik W. Frijlink, Barbro N. Melgert, Peter Olinga, and Wouter L.J. Hinrichs

SUPPLEMENTARY INFORMATION

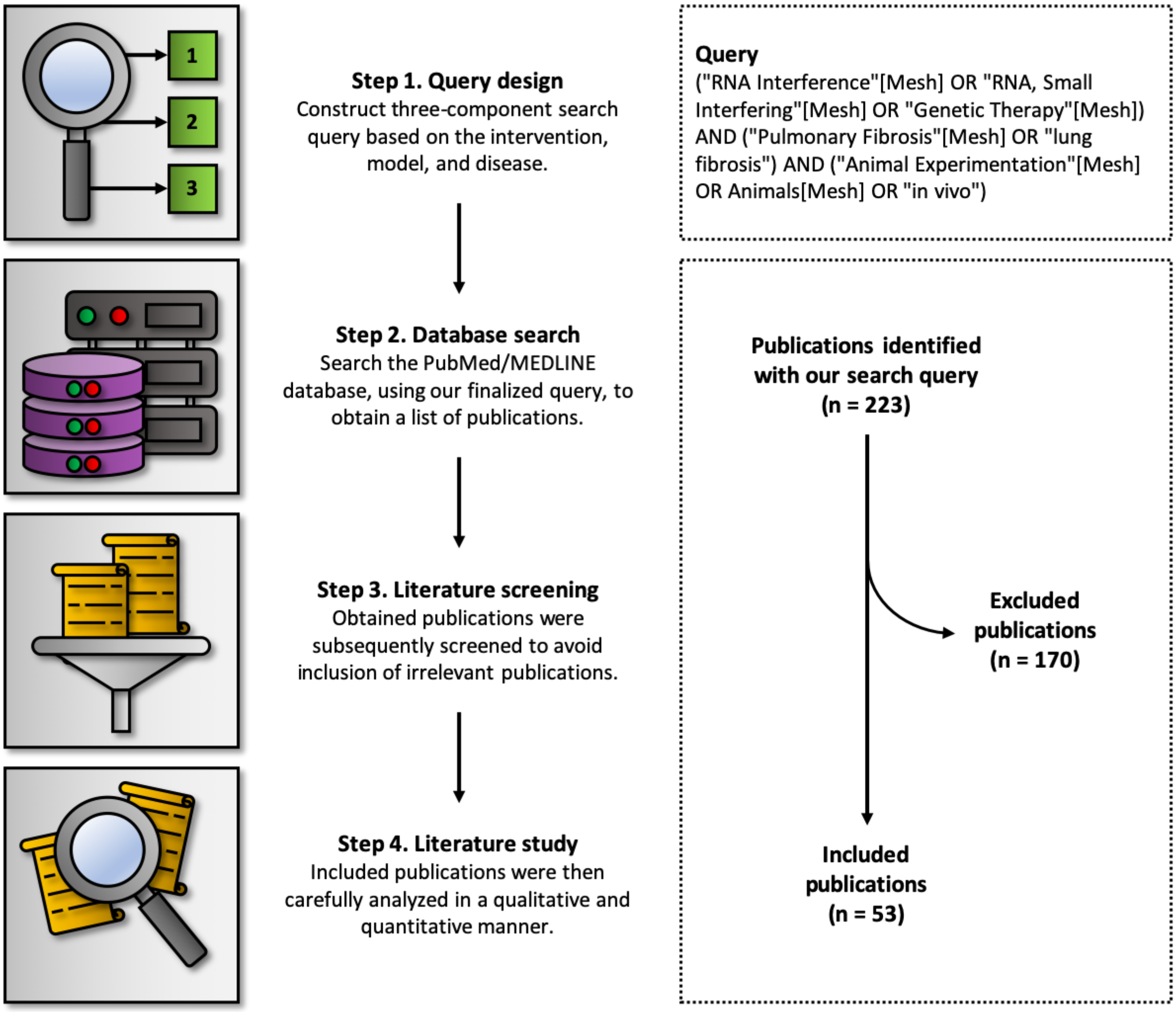

Supplemental Figure S1. Search strategy.

To find relevant publications, we conducted a literature study using the search strategy described in this figure. We first constructed a three-component search query based on the intervention (gene therapy), model (animal studies), and disease (pulmonary fibrosis). This search query was subsequently used to identify publications in the PubMed/MEDLINE database. The identified publications (n = 223) were screened to exclude publications that were not relevant (n = 170). Remaining publications (n = 53) were included in this review.

Supplemental Table S1. Nanoparticles.

This table summarizes information regarding the type and composition of nanoparticles used in cited studies. Unless not determined (n.d.), physiochemical properties are also described.

Concept	Component(s)	Cargo	Physicochemical properties	Ref.
Lipid-based	Lipofectin transfection reagent	pDNA	n.d.	[35]
Polymer-based	Macroaggregated albumin/polyethylenimine	pDNA	n.d.	[40]
Peptide-based	Hemagglutinating virus of Japan envelope proteins	siRNA	n.d.	[52]
Lipid-based	DharmaFect transfection reagent	siRNA	n.d.	[56]
Lipid-based	LipoTrust SR transfection reagent	siRNA	n.d.	[62]
Polymer-based	Poly(dimethylamino)ethylmethacrylate	siRNA	n.d.	[63]
Lipid-based	Conjugated siRNAs with lipid and polymer tails	siRNA	Z-average of ~100 nm and dispersity < 0.2	[67]
Lipid-based	Precirol and squalene	siRNA	Z-average of ~400 nm and neutral charge	[73]
Polymer-based	Polyethyleneimine	siRNA	n.d.	[78]
Peptide-based	Secondary amphipathic peptide of 20 residues	siRNA	n.d.	[79]
Polymer-based	Polyethyleneimine	pDNA	n.d.	[84]
